# Antipsychotic Prescribing to Patients Diagnosed with Dementia Without a Diagnosis of Psychosis in the Context of National Guidance and Drug Safety Warnings: Longitudinal Study in UK General Practice

**DOI:** 10.1007/s40264-017-0538-x

**Published:** 2017-04-24

**Authors:** S. Jill Stocks, Evangelos Kontopantelis, Roger T. Webb, Anthony J. Avery, Alistair Burns, Darren M. Ashcroft

**Affiliations:** 1 Division of Population Health, Health Services Research and Primary Care, School of Health Sciences, National Institute for Health Research Greater Manchester Primary Care Patient Safety Translational Research Centre, Centre for Primary Care, University of Manchester, Manchester, UK; 2 Division of Population Health, Health Services Research and Primary Care, School of Health Sciences, National Institute for Health Research School for Primary Care Research, Centre for Primary Care, University of Manchester, Manchester, UK; 3 Division of Informatics, Imaging and Data Sciences, School of Health Sciences, Centre for Health Informatics, University of Manchester, Manchester, UK; 4 Division of Psychology and Mental Health, School of Health Sciences, University of Manchester, Manchester, UK; 5 Division of Primary Care, School of Medicine, Queen’s Medical Centre, University of Nottingham, Nottingham, UK; 6 Division of Neuroscience and Experimental Psychology, School of Biological Sciences, University of Manchester, Manchester, UK; 7 Division of Pharmacy and Optometry, School of Health Sciences, Centre for Pharmacoepidemiology and Drug Safety, University of Manchester, Manchester Academic Health Sciences Centre, Manchester, UK

## Abstract

**Introduction:**

Policy interventions to address inappropriate prescribing of antipsychotic drugs to older people diagnosed with dementia are commonplace. In the UK, warnings were issued by the Medicines Healthcare products Regulatory Agency in 2004, 2009 and 2012 and the National Institute for Health and Care Excellence guidance was published in 2006. It is important to evaluate the impact of such interventions.

**Methods:**

We analysed routinely collected primary-care data from 111,346 patients attending one of 689 general practices contributing to the Clinical Practice Research Datalink to describe the temporal changes in the prescribing of antipsychotic drugs to patients aged 65 years or over diagnosed with dementia without a concomitant psychosis diagnosis from 2001 to 2014 using an interrupted time series and a before-and-after design. Logistic regression methods were used to quantify the impact of patient and practice level variables on prescribing prevalence.

**Results:**

Prescribing of first-generation antipsychotic drugs reduced from 8.9% in 2001 to 1.4% in 2014 (prevalence ratio 2014/2001 adjusted for age, sex and clustering within practices (0.14, 95% confidence interval 0.12–0.16), whereas there was little change for second-generation antipsychotic drugs (1.01, confidence interval 0.94–1.17). Between 2004 and 2012, several policy interventions coincided with a pattern of ups and downs, whereas the 2006 National Institute for Health and Care Excellence guidance was followed by a gradual longer term reduction. Since 2013, the decreasing trend in second-generation antipsychotic drug prescribing has plateaued largely driven by the increasing prescribing of risperidone.

**Conclusions:**

Increased surveillance and evaluation of drug safety warnings and guidance are needed to improve the impact of future interventions.

**Electronic supplementary material:**

The online version of this article (doi:10.1007/s40264-017-0538-x) contains supplementary material, which is available to authorized users.

## Key Points

**Table:** 

A Medicines Healthcare products Regulatory Agency drug safety warning in 2004 was associated with a marked short-term reduction in the prescribing of second-generation antipsychotic drugs to older patients with dementia, whereas subsequent Medicines Healthcare products Regulatory Agency warnings had little impact.
National Institute for Health and Care Excellence guidance published in 2006 was followed by a longer-term declining trend but was not temporally associated with an immediate reduction in prescribing.
Prescribing first-generation antipsychotic drugs to older patients with dementia without psychosis occurred much less frequently in 2014 than 2001 but prescribing of second-generation antipsychotic drugs remained similar.
A further, carefully worded, warning may be justified to reduce the longer-term prescribing of second-generation antipsychotic drugs to patients with dementia.

## Introduction

The risks of prescribing antipsychotic drugs to patients with dementia have been well documented [1–4]. Antipsychotic drugs may cause extrapyramidal side effects [5] and studies have shown that the newer second-generation atypical antipsychotic drugs are also associated with an increased risk of stroke [1, 6–9]. Regulatory bodies in several countries have issued warnings, guidance and advice aiming to reduce inappropriate prescribing of antipsychotic drugs to patients diagnosed with dementia [10–21]. It is important to evaluate the impact, and compare the methods of dissemination, of such interventions to inform future policy to reduce antipsychotic drug prescribing to patients with dementia as well as provide useful lessons on implementing national guidance and warnings.

In the UK, there have been several consecutive warnings and guidance aiming to reduce inappropriate prescribing of antipsychotic drugs to patients diagnosed with dementia (Table 1). During March 2004, the Committee for the Safety of Medicines (CSM) warned through a personal letter to all healthcare professionals marked ‘urgent’ that risperidone or olanzapine should not be used for the treatment of behavioural symptoms of dementia in older people because of a clearly documented increased risk of stroke [10]. Later studies, however, showed that similar risks existed for other second-generation antipsychotic drugs and indeed for first-generation antipsychotic drugs [8]. Guidelines published by the National Institute for Health and Care Excellence (NICE) in November 2006 recommended that antipsychotic drugs should not be prescribed for patients who experience behavioural and psychological symptoms of dementia of mild-to-moderate severity because of the increased risk of cerebrovascular adverse effects. This guideline stated that an antipsychotic drug should only be considered for patients with dementia with severe behavioural and psychological symptoms (psychosis and/or agitated behaviour causing significant distress), and only after other approaches have proved inadequate and the risks considered and discussed with the patient and/or their carers. Additionally, the prescription should be time limited and reviewed according to clinical need or every 3 months [11]. There is no legal obligation to follow NICE guidelines but their implementation is actively encouraged in England and Wales [12]. The approach to the implementation of NICE guidance varies between National Health Service organisations.

**Table 1:** Definition of dates for interrupted time series (ITS) analysis and before and after comparison of UK national interventions aiming to reduce the inappropriate prescribing of antipsychotic drugs to patients with dementia

Dates	Intervention period	Time periods for ITS analysis: 1. before intervention 2. During intervention 3. After intervention	Audit dates for before (1) and after comparison (2)
March 2004	MHRA Committee for the Safety of Medicines warning: risperidone and olanzapine should not be used to treat behavioural symptoms of dementia in older patients	1. March 2003 to February 2004 2. March 2004 to June 2004 3. July 2004 to June 2005	1. 1 March, 2004 2. 1 September 2004
November 2006	NICE guidelines: antipsychotic drugs should only be used for severe cognitive symptoms for a limited time after other approaches have proved inadequate	1. November 2005 to October 2006 2. November 2006 to February 2007 3. March 2007 to February 2008	1. 1 November, 2006 2. 1 May, 2007
March 2009	MHRA drug safety update: risperidone licenced for severe aggression in patients with Alzheimer’s disease and added to the MHRA’s Black Triangle list of medicines	1. March 2008 to February 2009 2. March 2009 to February 2010 3. March 2010 to February 2011	1. 1 March, 2009 2. 1 May, 2010
June 2009	Report on yellow card results and reiteration of the risks of antipsychotic drug use		
October 2009	Expert review called for urgent action		
November 2009	UK government pledge to reduce the use of antipsychotic drugs for patients with dementia		
March 2012	Prime minister launches the National Dementia Challenge	1. March 2011 to February 2012 2. March 2012 to August 2012 3. September 2012 to August 2013	1. 1 March, 2012 2. 1 November, 2012

*MHRA* Medicines Healthcare products Regulatory Agency, *NICE* National Institute for Health and Care Excellence

Following a 2008 review by the European Medicines Agency that aimed to harmonise the Summary of Product Characteristics for Risperdal^®^ (risperidone) [13], the UK Medicines Healthcare products Regulatory Agency (MHRA) released a drug safety update (DSU) in March 2009 stating that risperidone was licenced for the short-term (<6 weeks) treatment of severe aggression in people with Alzheimer’s disease. At the same time, risperidone was added to the MHRA’s Black Triangle list of medicines, indicating the start of intensive safety monitoring [14]. In June 2009, a further MHRA DSU reminded of the possible risks [15]. Drug safety updates are online e-bulletins published monthly on the UK Government website. Healthcare professionals can sign up to receive an email alert when the monthly update is published (http://www.medsiq.org/tool/drug-safety-update).

In October 2009, a review commissioned by the English Department of Health called for urgent action [16]. The report estimated that up to two-thirds of prescriptions of antipsychotic drugs to patients with dementia were unnecessary and potentially harmful. Following the report, the UK government pledged to reduce the use of antipsychotic drugs for people with dementia [17]. However, in May 2012, a MHRA DSU noted that, despite an encouraging overall reduction in the proportion of elderly people with dementia being prescribed antipsychotic drugs since 2007, the reductions fell short of the hoped-for levels [18]. The UK government has continued to place a high priority on reducing the inappropriate prescribing of antipsychotic drugs for people with dementia; the Prime Minister launched a national challenge to fight dementia in March 2012 followed by the ‘Challenge on Dementia 2020’ in 2015 [17, 19]. Reducing inappropriate prescribing of antipsychotic drugs to patients with dementia is a specified aim in this national initiative. A recent study estimated that around 8% of patients aged 65 years or over with a dementia diagnosis received a potentially inappropriate prescription for an antipsychotic drug [22].

The impact of these UK interventions aiming to reduce the inappropriate prescribing of antipsychotic drugs to older people with dementia has been investigated in Scottish and UK general practice [23–26]. Both studies found that the 2004 warning was associated with an immediate but short-term reduction in antipsychotic drug prescribing, whereas the 2009 intervention was not associated with an immediate reduction but was followed by a declining trend in antipsychotic drug prescribing. Furthermore, both studies highlighted that the 2004 decline was driven largely by a reduction in the prescribing of risperidone and olanzapine, but there was a subsequent increase in the prescribing of quetiapine [24]. Another interrupted time series (ITS) study of the prescribing of second-generation antipsychotic drugs in one National Health Service Hospital Foundation Trust also found a decline following the 2004 warning and increasing use of risperidone following the 2009 warning [27]. Furthermore, a national audit observed a decline in the prescribing of antipsychotic drugs to patients with dementia from 1995 to 2011, [18] subsequently confirmed by a more recent study using anonymised primary care data [28]. These studies [18, 28] considered overall antipsychotic drug prescribing but did not consider different classes or specific antipsychotic drugs in detail.

The prescribing of antipsychotic drugs to patients with dementia in Italy, USA and Canada has also been examined in the context of policy interventions. In Italy, a similar warning to the 2004 CSM warning coincided with a short-lived reduction in antipsychotic drug prescribing, particularly second generation, but from 2009 there was an increasing trend for both classes of antipsychotic drug [26]. During 2005, the US Food and Drug Administration issued a warning concerning the use of atypical antipsychotic drugs in dementia that was found to be associated with a reduction in prescribing recorded in the national Veterans Affairs dataset [20]. A Canadian study found a deceleration in the growth of atypical antipsychotic drug use among patients with dementia, but no overall reduction in prescribing [21].

These policy interventions aim to reduce inappropriate prescribing rather than simply reduce prescribing, thus it is important to use clinically meaningful measures. Previously, we have used prescribing safety indicators to address potentially inappropriate prescribing [22, 29]. In this study, we have used a prescribing safety indicator (PSI) that specifically addresses the prescribing of antipsychotic drugs to patients with dementia using anonymised, patient-level electronic health records from the Clinical Practice Research Datalink (CPRD) [30] to investigate the changes in prescribing in the context of these policy interventions. Our aims were to: (1) examine changes in the prevalence of repeated prescribing of antipsychotic drugs (for at least 3 months) to patients aged 65 years or over diagnosed with dementia and without a psychosis diagnosis from 2001 to 2014; (2) compare the proportion of older people with dementia without a psychosis diagnosis prescribed a repeated antipsychotic drug before and after the policy interventions (warnings, updates, guidelines, reports) described above; (3) analyse the changes in monthly prescribing of antipsychotic drugs to older patients with dementia without a psychosis diagnosis before, during and after the interventions using an ITS design; and (4) examine the patient- and practice-level predictors of prescribing antipsychotic drugs to patients aged 65 years or over with dementia without a psychosis diagnosis and the variability between practices.

## Methods

### Population and Coding of Diagnoses and Prescriptions

The CPRD is a primary care database of anonymised electronic health records from general practice that includes around 7% of the UK population and is broadly representative of the UK population in terms of age, sex and ethnicity [30]. All patients aged 65 years or over (defined by the year when they turned 66) registered with one of the CPRD practices between 1 January, 2001 and 31 December, 2014 that had uploaded data of research quality on or after the audit date were included in the analysis. Patients were only included from the time they registered with a CPRD practice but their relevant diagnoses (dementia and psychosis) could have been recorded before their most recent registration date as their records would have been transferred from their previous practice. Patients were categorised according to codes recorded in their primary care record describing the diagnosis of dementia or psychosis and the prescribing of antipsychotic drugs as described elsewhere [22]. A full list of codes to describe the diagnoses of dementia, psychosis and antipsychotic drugs prescribed is provided in Electronic Supplementary Material 1 and has been uploaded to https://clinicalcodes.rss.mhs.man.ac.uk/medcodes/article/53/ [31].

### Cross-Sectional Estimates of the Prevalence of Potentially Inappropriate Prescribing of Antipsychotic Drugs

The proportion of patients diagnosed with dementia and without a psychosis diagnosis who were prescribed an antipsychotic drug was estimated using a PSI as described previously [22]. The PSI denominator included all patients aged 65 years or over with dementia without a psychosis diagnosis 6 months prior to an audit date. The PSI numerator included all patients prescribed an antipsychotic drug (by class and specific antipsychotic drug) at least twice during the 6 months leading up to an audit date and with at least 3 months between the first and last prescription. The associations between patient and practice level predictors and the prevalence of the PSI with an audit date of 31 December, 2014 was estimated using a mixed-effects, two-level logistic regression model with random effects at the practice level (i.e., patients nested within practices) in Stata Version 14 (StataCorp, College Station, Texas, USA). The practice-level predictors were list size, practice location by region, index of multiple deprivation quintile and the proportion of patients aged 65 years or older registered at the practice (practices with a higher proportion of older patients are more likely to be providers of care for residential homes). Patient-level predictors were age, sex and polypharmacy; polypharmacy was defined as the number of drugs with at least two prescriptions of the same drug on different dates (excluding antipsychotic drugs) within the 12 months leading up to the audit date. The heterogeneity between practices for the PSI was quantified by the 95% prediction intervals derived from the model with 95% confidence as described elsewhere [32].

### Longitudinal Changes in Prescribing in the Context of Warnings and Guidance

**Fig. 1:**
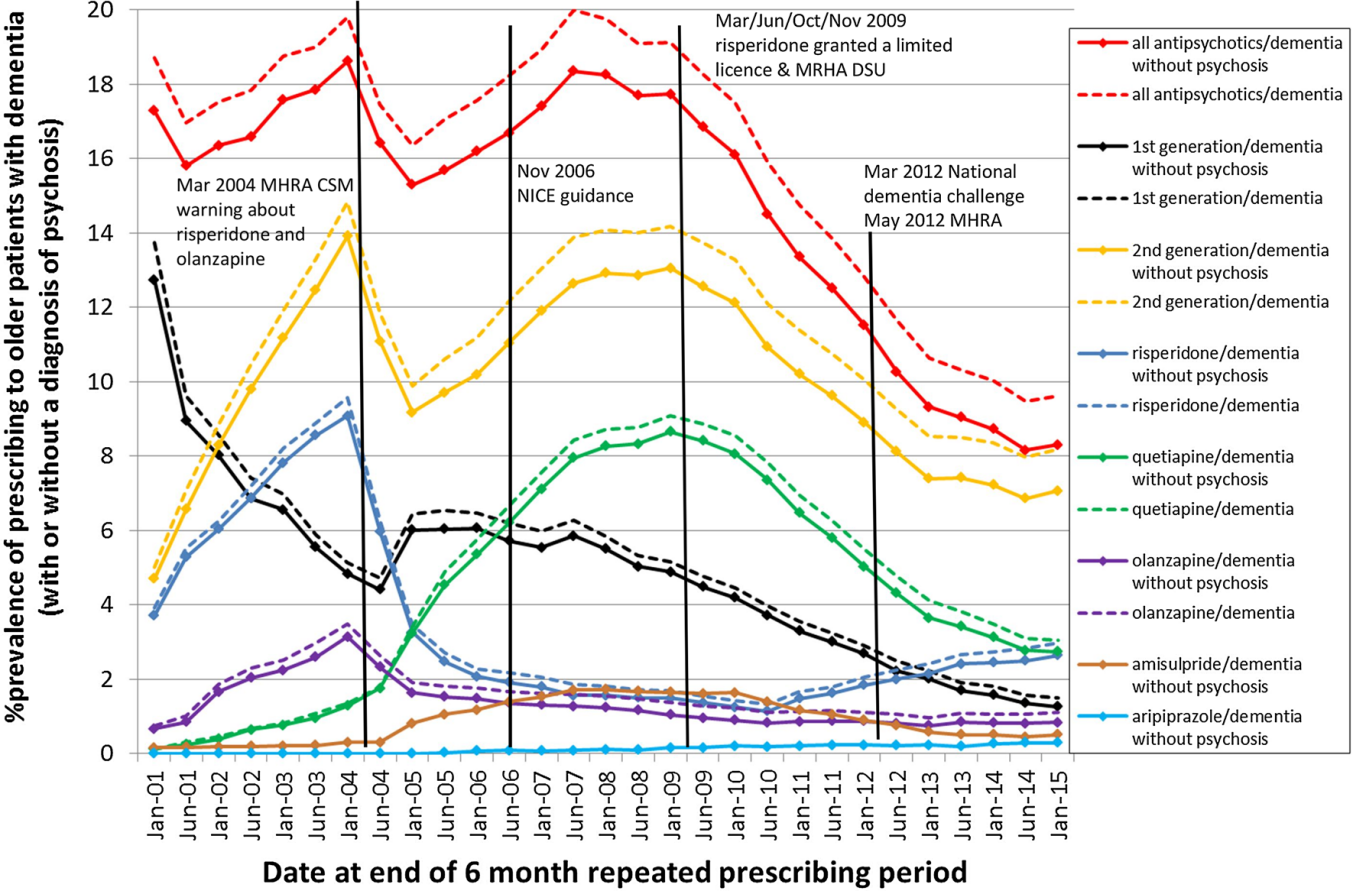
Prevalence of prescribing repeated antipsychotic drugs to older patients diagnosed with dementia and without a psychosis diagnosis during the 6 months leading up to the audit date alongside the dates of interventions. *Dashed lines* indicate prevalence for all patients with dementia (with or without a psychosis diagnosis)

Using two different approaches, we investigated temporal changes in prescribing. First, a series of consecutive cross-sectional analyses were undertaken using the PSI method described above with a 6-monthly rolling time window of audit dates from 2001 to 2014 (Fig. 1). To specifically investigate the change in the prevalence of the PSI following the interventions, two audit dates covering the 6 months leading up to, and following, the date of the intervention(s) were compared as a prevalence ratio (Table 1). This before-and-after analysis used the two-level logistic regression model described above that also included a categorical variable coding for the time periods before and after the interventions (Table 1). This analysis allows for the clinically appropriate short-term prescribing, as described in the 2006 NICE guidelines [11], by excluding prescribing occurring for less than 3 months from the numerator and for ‘end of life’ prescribing by excluding patients who died within the 6 months leading up to the audit date from the denominator. However, it does not take into account the pre- and post-intervention trends. Therefore, a second approach used an ITS design, which accounts for trends, to compare monthly changes in prescribing within consecutive time periods. The time periods were defined prospective to the analysis with respect to the interventions described in Sect. 1 (Table 1) and represented the time 12 months before, the month of the intervention, and the following 3 months (during period) and 12 months after each intervention. The ‘during’ period was to allow time for the information to be disseminated to practices and actioned. When interventions occurred closely in time, all interventions were included in the ‘during’ period. The period of 12 months before and after the intervention was judged to provided sufficient observation points to measure the secular time trend and minimise the effect of seasonality (e.g., holidays). However, keeping the time periods temporally close to the intervention reduces potential confounding by events occurring at a more distant time. Sensitivity analyses repeating these longitudinal analyses using a cohort of all patients with dementia (including those with a psychosis diagnosis) and descriptive analyses of the temporal changes in the study population are described in Electronic Supplementary Material 2.

For the ITS analysis, one prescription was sufficient for inclusion in the numerator, rather than two prescriptions at least 3 months apart as described for the PSI analyses. This ensured no overlap between the time periods of observation, a prerequisite for ITS. The statistical comparison between trends in monthly prescribing in different time periods took the autocorrelation of the residuals into account by using an autoregressive, integrated moving average model with a lag of 1 month [33]. In addition, ordinary least-squares regression was used to estimate the temporal trends before, during and after the interventions with Newey–West standard errors to handle autocorrelation. The ITS used the itsa command in Stata but the two-level version of the model could not be used as some practices did not contribute to the CPRD continuously from 2003 to 2013, thereby creating an unbalanced data structure.

## Results

Data from 111,346 patients aged 65 years or older with a diagnosis of dementia and without psychosis, attending 689 UK general practices contributing data to the CPRD for at least 6 months between 1 January, 2001 and 31 December, 2014, were analysed (median number of practices included per year 573; range 459–601). The temporal changes in the study population from 2001 to 2014 are described in Electronic Supplementary Material 2.

### Temporal Changes in Prescribing of Antipsychotic Drugs from 2001 to 2014

**Fig. 2:**
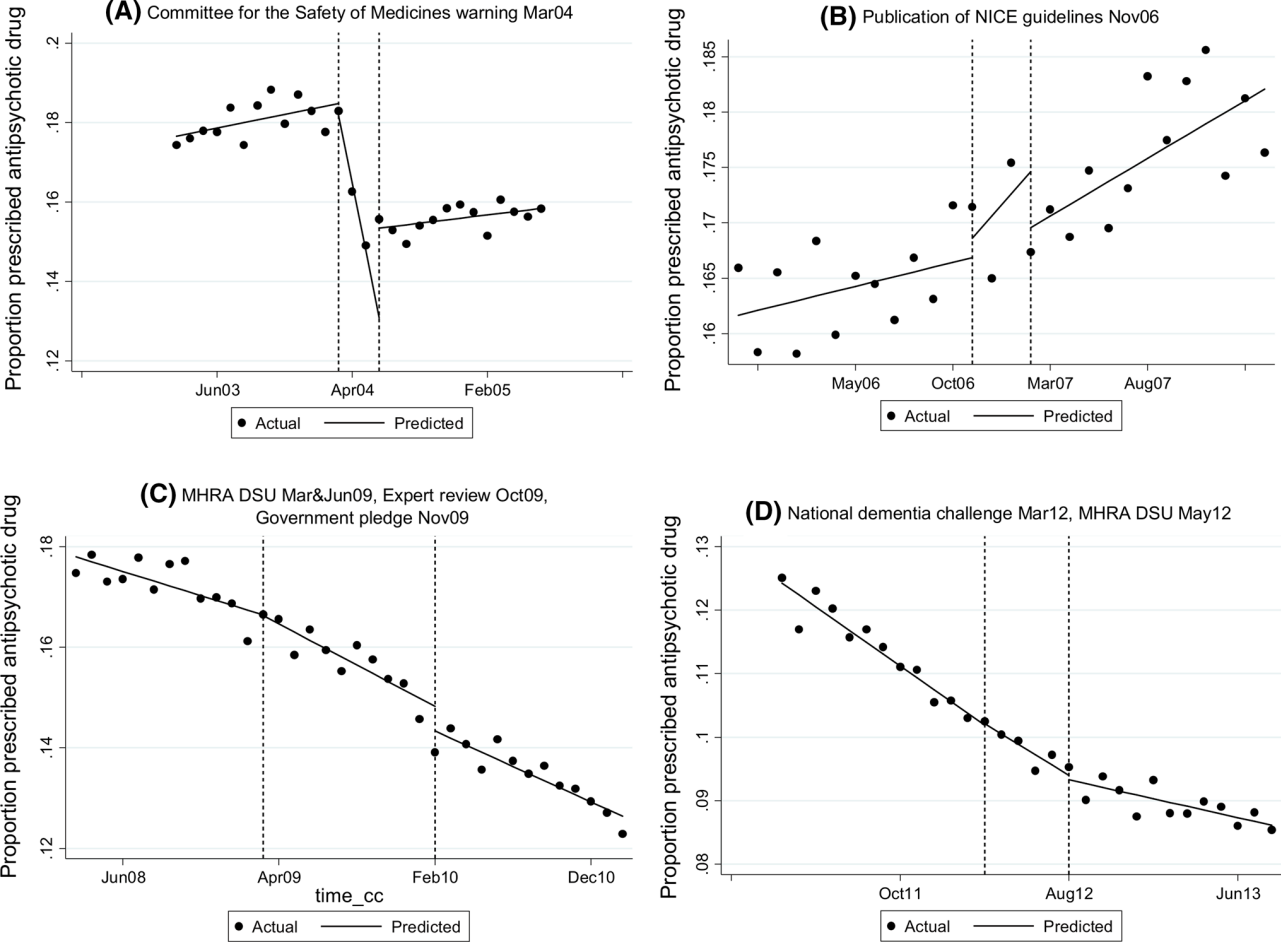
Interrupted time series analyses for monthly prescribing of antipsychotic drugs to older patients diagnosed with dementia and without a psychosis diagnosis; boundary points defined according to warnings and guidance described in Table 1

Figure 1 shows the proportion of patients prescribed antipsychotic drugs at least twice with at least 3 months between the first and last prescription during the 6 months leading up to an audit date (numerator) out of all patients aged 65 years or over diagnosed with dementia, with or without a psychosis diagnosis (denominator), alongside the dates of the warnings and advice (Table 1). From 2001 to 2014, the observed proportion of patients receiving repeated prescriptions for an antipsychotic drug fell from 15.8 to 8.2%. The majority of the reduction was the result of decreased prescribing of first-generation antipsychotic drugs, which fell from 8.9 to 1.4%. The opposite was observed for second-generation antipsychotic drugs where the observed proportion was similar in 2001 at 6.6% and 2014 at 6.9%. The corresponding prevalence ratios for repeated prescribing at audit date 1 June, 2014 relative to 1 June, 2001 from the two-level logistic regression model with random effects at the practice level and adjusted for age and sex were 0.48 (95% CI 0.44–0.52) for all antipsychotic drugs; 1.01 (95% CI 0.94–1.17) for second-generation antipsychotic drugs and 0.14 (95% CI 0.12–0.16) for first-generation antipsychotic drugs. During 2003 and 2007–8, however, the observed repeated prescribing prevalence of second-generation antipsychotic drugs peaked at around 18%, significantly higher than the 2014 prevalence [prevalence ratio for audit date 1 June, 2014 adjusted for age and sex relative to (1) 1 January, 2004: 0.38, 0.36–0.41; and (2) 1 January, 2008: 0.41, 0.38–0.43]. The results from the dementia cohort without excluding patients with a psychosis diagnosis (sensitivity analysis) closely agreed with the results from the main analysis (dashed lines in Fig. 1). The estimates of the monthly change in prescribing of at least one antipsychotic drug before, during and after each intervention are shown in Table 2 alongside the *p* values comparing the monthly change before the intervention to during and after the intervention (the ITS analysis). Plots depicting the ITS analysis (corresponding to Table 2) are shown for all antipsychotic drugs in Fig. 2 and according to the class and specific drug in Electronic Supplementary Material 3. Table 2 also shows the estimates of the change in prevalence of repeated prescribing during the 6 months before and after each intervention as prevalence ratios.

**Table 2:** Temporal changes in the prescribing of antipsychotic drugs at the time of the national interventions described in Table 1

Drug type	Predicted proportion prescribed 12 months before^c^ (%)	Predicted monthly change in proportion prescribed (95% CI)^a^, *p* value^b^			Predicted proportion prescribed 12 months after^c^ (%)	Prevalence ratio before/after (95% CI)^d^
		12 months before intervention	During intervention (see Table 1)	12 months following ‘during’ the intervention period		
UK MHRA Committee for the Safety of Medicines, warning March 2004						
All antipsychotic drugs	17.6	0.07 (−0.00 to 0.14)	−1.69 (−1.88 to −1.51) P_D_ < 0.001	0.04 (0.00–0.08) P_A_ < 0.001	15.8	0.81 (0.76–0.87)
First-generation antipsychotic drugs	5.5	−0.12 (−0.14 to −0.10)	0.40 (0.27 to 0.53) P_D_ < 0.001	0.03 (0.01–0.05) P_A_ < 0.001	6.5	1.17 (1.03–1.31)
Second-generation antipsychotic drugs	12.6	0.19 (0.13–0.23)	−2.27 (−2.59 to −1.95) P_D_ < 0.001	0.01 (−0.01 to 0.04) P_A_ < 0.001	9.7	0.66 (0.61–0.71)
Risperidone	8.9	0.04 (0.01–0.07)	−1.98 (−2.36 to −1.61) P_D_ < 0.001	−0.17 (−0.23 to −0.12) P_A_ < 0.001	1.9	0.48 (0.43–0.53)
Olanzapine	2.6	0.08 (0.06–0.10)	−0.62 (−0.65 to −0.58 P_D_ < 0.001	−0.04 (−0.05 to −0.03) P_A_ < 0.001	1.3	0.60 (0.51–0.70)
Quetiapine	0.9	0.06 (0.05–0.07)	0.24 (0.18–0.30) P_D_ < 0.001	0.18 (0.14–0.22) P_A_ = 0. 17	5.2	1.51 (1.24–1.83)
Amisulpride	0.3	0.01 (0.00 to 0.01)	0.07 (0.04–0.11) P_D_ < 0.001	0.04 (0.03–0.05) P_A_ = 0.05	1.27	1.47 (1.00–2.15)
NICE guidelines, November 2006						
All antipsychotic drugs	16.2	0.04 (0.00–0.09)	0.20 (−0.26 to 0.67) P_D_ = 0. 51	0.10 (0.04–0.16) P_A_ = 0.67	14.1	1.07 (1.01–1.13)
First-generation antipsychotic drugs	6.2	−0.06 (−0.08 to 0.03)	0.03 (−0.16 to 0.21) P_D_ = 0. 40	0.00 (−0.02 to 0.01) P_A_ = 0.75	5.6	1.02 (0.93–1.12)
Second-generation antipsychotic drugs	10.3	0.10 (0.07 to 0.13)	0.21 (−0.09 to 0.52) P_D_ = 0. 46	0.11 (0.07–0.16) P_A_ = 0.52	13.0	1.08 (1.01–1.15)
Risperidone	2.0	−0.03 (−0.04 to −0.02)	−0.01 (−0.06 to 0.03) P_D_ = 0. 61	0.00 (−0.00 to 0.01) P_A_ = 0.43	1.6	0.87 (0.74–1.03)
Olanzapine	1.3	−0.01 (−0.01 to 0.01)	0.02 (−0.03 to 0.07) P_D_ = 0. 25	−0.00 (−0.01 to 0.01) P_A_ = 0.45	0.9	0.97 (0.80–1.17)
Quetiapine	5.6	0.12 (010–0.13)	0.21 (−0.02 to 0.43) P_D_ = 0. 43	0.10 (0.07–0.13) P_A_ = 0.34	6.6	1.14 (1.05–1.24)
Amisulpride	1.0	0.02 (0.01–0.03)	0.00 (−0.01 to 0.002) P_D_ = 0. 03	0.01 (0.01–0.01) P_A_ = 0.38	1.4	1.10 (0.92–1.31)
UK MHRA drug safety updates, March and June 2009, expert review October 2009, government pledge November 2009						
All antipsychotic drugs	17.8	−0.09 (−0.21 to −0.03)	−0.16 (−0.21 to −0.12) P_D_ = 0.08	−0.14 (−0.18 to −0.01) P_A_ = 0.53	12.6	0.83 (0.79–0.88)
First-generation antipsychotic drugs	5.2	−0.05 (−0.07 to −0.03)	-0.07 (−0.08 to −0.05) P_D_ = 0.10	−0.05 (−0.06 to −0.05) P_A_ = 0.13	3.2	0.85 (0.77–0.95)
Second-generation antipsychotic drugs	12.9	−0.05 (−0.10 to −0.00)	−0.10 (−0.14 to −0.06) P_D_ = 0.12	−0.09 (−0.13 to −0.06) P_A_ = 0.80	9.7	0.84 (0.79–0.90)
Risperidone	1.6	−0.02 (−0.02 to −0.01)	−0.02 (−0.02 to −0.01) P_D_ = 0.52	0.05 (0.04–0.06) P_A_ < 0.001	1.8	0.73 (0.61–0.87)
Olanzapine	1.1	−0.02 (−0.03 to −0.01)	−0.01 (−0.02 to −0.00) P_D_ = 0.19	0.00 (−0.00 to 0.01) P_A_ = 0.005	0.8	0.81 (0.66–1.00)
Quetiapine	8.4	0.01 (−0.04 to 0.03)	−0.07 (−0.11 to −0.04) P_D_ = 0.008	−0.12 (−0.14 to −0.09) P_A_ = 0.07	5.8	0.87 (0.81–0.94)
Amisulpride	1.8	−0.02 (−0.02 to −0.01)	0.00 (−0.00 to 0.01) P_D_ < 0.001	−0.03 (−0.04 to −0.03) P_A_ < 0.001	1.0	0.92 (0.78–1.09)
National Dementia Challenge March 2012, MHRA drug safety update May 2012						
All antipsychotic drugs	12.4	−0.19 (−0.22 to −0.15)	−0.16 (−0.23 to −0.09) P_D_ = 0.54	−0.06 (−0.08 to −0.04) P_A_ = 0.01	8.6	0.86 (0.81–0.92)
First-generation antipsychotic drugs	3.1	−0.06 (−0.07 to −0.05)	−0.03 (−0.06 to −0.01) P_D_ = 0.05	−0.05 (−0.05 to −0.04) P_A_ = 0.39	1.7	0.81 (0.71–0.91)
Second-generation antipsychotic drugs	9.4	−0.13 (−0.15 to −0.10)	−0.13 (−0.19 to −0.08) P_D_ = 0.84	−0.01 (−0.03 to −0.00) P_A_ < 0.001	7.1	0.89 (0.83–0.95)
Risperidone	1.8	0.02 (0.01–0.03)	0.02 (0.00–0.04) P_D_ = 0.87	0.03 (0.02–0.04) P_A_ = 0.30	2.6	1.13 (0.99–1.29)
Olanzapine	0.8	−0.01 (−0.01 to −0.00)	−0.01 (−0.01 to 0.00) P_D_ = 0.71	−0.00 (−0.01 to 0.00) P_A_ = 0.30	0.7	0.93 (0.76–1.14)
Quetiapine	5.6	−0.11 (−0.13 to −0.09)	−0.12 (−0.14 to −0.10) P_D_ = 0.34	−0.04 (−0.05 to −0.03) P_A_ < 0.001	3.0	0.80 (0.74–0.88)
Amisulpride	1.0	−0.03 (−0.03 to −0.02)	−0.02 (−0.03 to −0.02) P_D_ = 0.54	−0.01 (−0.01 to −0.00) P_A_ = 0.004	0.5	0.80 (0.64–0.99)

*CI* confidence interval, *MHRA* Medicines Healthcare products Regulatory Agency, *NICE* National Institute for Health and Care Excellence

^a^Predicted monthly change is the absolute value not relative to the starting prevalence, e.g., for row 1 the starting prevalence is 17.6%, which increases by 0.08 per month making a value of 17.68% in the following month, assuming a linear trend (for illustration only, the method does not assume a linear trend)

^b^ *p* value tests null hypothesis that there is no difference in the monthly change in receiving at least one prescription before and after the intervention using a cut-off point of the start of the intervention period (i.e., P_D_ compares ‘during and after’ with ‘before’) or using a cut-offpoint of the end of the intervention period *(*i.e., P_A_ compares ‘after’ with ‘before and during’)

^c^Predicted proportion (%) of patients prescribed at least one antipsychotic drug during the month exactly 12 months before the intervention or at the end of the observation period (Table 1)

^d^Prevalence ratio compares repeated prescribing for 3 months or longer during the 6 months after the intervention relative to the 6 months leading up to the intervention as defined in Table 1

### Temporal Changes in the Context of Policy Interventions

During the 6 months following the 2004 CSM warning, there was a marked decline in the prevalence of repeated prescribing of second-generation antipsychotic drugs (0.66; 0.61–0.71, Table 2) and an increase for first-generation antipsychotic drugs (1.17; 1.03–1.31, Table 2). The decline in prescribing of second-generation antipsychotic drugs was driven mainly by reductions in prescribing of risperidone and olanzapine but was attenuated by an increase in the prescribing of quetiapine and amisulpride (see Panel A, Electronic Supplementary Material 3). The pre-warning increasing trend in monthly prescribing of second-generation antipsychotic drugs flattened following the warning, whereas the pre-warning decreasing trend for first-generation antipsychotic drugs reversed to a post-warning increasing trend (see Panel A, Electronic Supplementary Material 3). Between the 2004 CSM warning and the 2006 NICE guidance, the prescribing trends had returned to the pre-CSM direction (i.e., increasing second generation and decreasing first generation, see Panel B, Electronic Supplementary Material 3). There were no significant changes in prescribing during or immediately following the 2006 NICE guidelines or in the prevalence of repeated prescribing during the 6 months following the publication of the guidelines (Table 2). The prescribing of second-generation antipsychotic drugs plateaued during 2007 and 2008, over a year after the publication of the 2006 NICE guidance (Fig. 1), then began to decline.

Around the same time, the prescribing of first-generation antipsychotic drugs began to decline more steeply. Prior to the series of interventions during 2009 (MHRA DSU: March and June; expert review: October; Government pledge: November) (Table 1), there was a consistent declining trend in prescribing for both classes of antipsychotic drug that steepened for second-generation antipsychotic drugs (see Panel C, Electronic Supplementary Material 3) but this was not significant (Table 2). An increasing trend in risperidone prescribing commenced around February 2010, approximately 11 months after the March 2009 DSU stated it was licensed for short-term use (see Panel C, Electronic Supplementary Material 3). The decline in prevalence for risperidone even though there is a post-intervention increasing trend (Table 2) reflects the a priori selection of the time periods (Table 1). Following the March 2012 National Dementia Challenge and May 2012 MHRA DSU, the decreasing trend in prescribing of second-generation antipsychotic drugs including olanzapine, quetiapine and amisulpride began to plateau but risperidone continued along an upward trajectory (see Panel D, Electronic Supplementary Material 3). During 2013, the downward trend in prescribing second-generation antipsychotic drugs plateaued driven largely by the increasing trend for risperidone prescribing (see Fig. 1 and Panel D in Electronic Supplementary Material 3). The autoregressive, integrated moving average model predicted that 7.2% of patients were prescribed a second-generation antipsychotic drug during January 2013, which remained similar over the next 2 years (the predicted monthly change from January 2013 to December 2014 was −0.01% (−0.01 to 0.00%).

### Cross-Sectional Analysis of Repeated Antipsychotic Prescribing between June and December 2014

The prevalence of patients with a diagnosis of dementia but not psychosis and a repeated prescription for an antipsychotic drug by patient and practice characteristics, as well as unadjusted and adjusted odds ratios derived from the multilevel mixed effects logistic regression model are shown in Table 3. The variation in prescribing between practices was high (see Figure C, Electronic Supplementary Material 2); the adjusted prediction intervals (expected range of prevalence for a new practice joining the analysis) for the time period January to June 2014 were 2.5–17.3%.

**Table 3:** Prevalence of patients with dementia without a psychosis diagnosis receiving repeated antipsychotic drug prescriptions between 1 January, and 30 June, 2014 and unadjusted and adjusted odds ratios from a two-level logistic regression model with random effects at the practice level

Variable	Observed prevalence (%)	Unadjusted odds ratio (95% CI)	Adjusted odds ratio (95% CI)
Age, years			
65–70	138/1181 (11.7)	1 (reference)	1 (reference)
71–75	232/2236 (10.4)	0.88 (0.70–1.11)	0.86 (0.68–1.09)
76–80	350/4007 (8.7)	0.73 (0.59–0.91)	0.70 (0.56–0.87)
81–85	448/5764 (7.8)	0.64 (0.52–0.79)	0.61 (0.49–0.75)
>85	712/9453 (7.5)	0.62 (0.51–0.75)	0.58 (0.48–0.71)
Number of medications on repeat prescriptions in the previous 12 months (excluding antipsychotic drugs)			
0–5	439/7519 (5.8)	1 (reference)	1 (reference)
6–9	472/6121 (7.7)	1.47 (1.27–1.71)	1.47 (1.27–1.70)
10–14	547/5450 (10.0)	1.94 (1.68–2.25)	1.96 (1.69–2.26)
15 or more	422/3551 (11.9)	2.33 (2.00–2.73)	2.31 (1.98–2.70)
Sex			
Male	647/7857 (8.2)	1 (reference)	1 (reference)
Female	1233/14784 (8.3)	1.00 (0.90–1.11)	1.06 (0.95–1.17)
List size (quartiles)			
<5000	202/2438 (8.3)	1 (reference)	1 (reference)
5000 to <8000	440/5197 (8.5)	1.06 (0.8–1.38)	0.96 (0.75–1.22)
8000 to <11,000	587/7253 (8.1)	1.04 (0.80–1.35)	0.97 (0.76–1.24)
>11,000	651/7753 (8.4)	1.07 (0.82–1.39)	1.10 (0.86–1.42)
Proportion (%) of patients in practice aged 65 years or over (CPRD mean = 19.6%)			
<15	289/3409 (8.5)	1 (reference)	1 (reference)
15 to <20	733/8181 (9.0)	1.14 (0.90–1.45)	1.04 (0.83–1.31)
20 to <25	632/7565 (8.4)	1.06 (0.83–1.36)	0.95 (0.75–1.21)
>25	226/3486 (6.5)	0.84 (0.61–1.15)	0.82 (0.60–1.12)
Practice level index of multiple deprivation			
1 least deprived	409/4801 (8.5)	1 (reference)	1 (reference)
2	415/4578 (9.1)	1.07 (0.82 1.40)	1.24 (0.97–1.59)
3	386/4723 (8.2)	0.99 (0.76–1.29)	1.16 (0.90–1.49)
4	371/4767 (7.8)	1.04 (0.80–1.36)	1.09 (0.85–1.40)
5 most deprived	299/3772 (7.9)	0.99 (0.75–1.30)	0.96 (0.73–1.25)
Region			
North West	204/2309 (8.8)	1 (reference)	1 (reference)
North East	6/202 (3.0)	0.29 (0.10–0.82)	0.30 (0.11–0.86)
Yorkshire/Humber	15/251 (6.0)	0.69 (0.31–1.53)	0.71 (0.32–1.59)
West Midlands	175/1994 (8.8)	0.96 (0.68–1.35)	1.05 (0.74–1.50)
East of England	126/1380 (9.1)	1.00 (0.69–1.45)	1.06 (0.72–1.56)
South West	110/1888 (5.8)	0.58 (0.40–0.84)	0.61 (0.41–0.90)
South central	203/3270 (6.2)	0.63 (0.46–0.87)	0.64 (0.46–0.90)
London	114/2017 (5.7)	0.57 (0.40–0.80)	0.56 (0.39–0.80)
South East coast	155/2506 (6.2)	0.59 (0.43–0.83)	0.63 (0.45–0.89)
Northern Ireland	166/1137 (14.6)	1.74 (1.20 to 2.53)	1.51 (1.03–2.22)
Scotland	342/3516 (9.7)	1.07 (0.80 to 1.42)	1.14 (0.85–1.53)
Wales	264/2171 (12.2)	1.43 (1.05 to 1.94)	1.55 (1.13–2.11)

*CI* confidence interval, *CPRD* Clinical Practice Research Datalink

In the 6 months leading up to 30 June, 2014 younger patients (aged 65–75 years) and those taking multiple medications were more likely to be prescribed antipsychotic drugs (Table 3). None of the practice-level variables or sex significantly affected the likelihood of being prescribed repeated antipsychotic drugs (list size, proportion of patients aged 65 years or over, index of multiple deprivation based on the practice postcode, Table 3). There were some differences between geographical regions of the UK with prescribing in Wales and Northern Ireland being more frequent than regions in the south of England.

## Discussion

Our most important finding is that the prescribing of first-generation antipsychotic drugs to older patients diagnosed with dementia and without a psychosis diagnosis declined substantially between 2001 and 2014. The frequency of prescribing second-generation antipsychotic drugs, however, despite a pattern of ups and downs, was essentially the same in 2014 as in 2001. Since 2013, the declining trend in second-generation antipsychotic prescribing has been halted, driven almost exclusively by the increasing prescribing of risperidone.

### Prescribing Patterns in the Context of National Guidelines and Drug Safety Warnings

Fluctuations in the prevalence of prescribing antipsychotic drugs and switching between classes and specific antipsychotic drugs were generally consistent with clinicians responding to national guidelines and drug safety warnings. The most noticeable change in prescribing, however, did not temporally coincide with any specific policy intervention. This was a plateauing of the increasing trend for second-generation antipsychotic drugs towards the end of 2007 and the commencement of a decreasing trend during 2008. The most recent preceding intervention was the publication of the 2006 NICE guidance and possibly this had a more gradual and long-term impact compared with the earlier 2004 CSM warning.

The large changes in prescribing coinciding with the 2004 CSM warning suggest that this type of direct warning can persuade clinicians to change their prescribing habits and place a responsibility on the issuer to ensure that the warning does not have unintended consequences. For example, specifying risperidone and olanzapine in the warning may have precipitated the increased prescribing of first-generation antipsychotic drugs as well as quetiapine and amisulpride. Similarly, specifying risperidone in the March 2009 DSU was followed by an increase in its prescribing some months later. Some increase in monthly prescribing might be expected given that the March 2009 DSU pointed out that risperidone was licenced specifically for short term (‘up to 6 weeks’) treatment of persistent aggression in Alzheimer’s dementia, but it should not have resulted in the observed increased prevalence of repeated prescribing for 3 months or longer.

The more marked effect of the 2004 CSM warning compared with the 2009 MHRA DSU has been observed previously in the UK [24, 26]. As pointed out by these authors [24, 26], the nature of the warnings might explain these large differences in effect. The 2004 CSM warning was sent to all healthcare professionals marked ‘urgent message’’ and contained explicit and clear guidance on how prescribers should respond [10], whereas the 2009 MHRA DSU simply recommended caution in initiation through a limited circulation bulletin [14]. We further suggest that the impact of the NICE guidance was more gradual and sustained in its impact, potentially reflecting different implementation policies and priorities across NHS trusts and general practices. We had decided a priori that 6 or 12 months following the warning or guidance was an appropriate length of time to judge whether or not an intervention had been effective but it is also important to look at the longer-term impact of consecutive warnings.

### Variation in Prescribing Patterns between Practices and Patients

The wide variation in prescribing between practices, even after adjusting for patient- and practice-level predictors, is of interest. The reliability of these prescribing safety indicator prevalence estimates has been discussed in a previous study; around 80% of the practices have sufficient patients aged over 65 years with a diagnosis of dementia and without psychosis to allow valid comparisons between practices [22]. The lower levels of prescribing to patients aged over 75 years may reflect advice provided by the Royal College of Psychiatrists to accompany the 2004 CSM warning that highlighted the increased risks for older patients [34]. It is not clear why multi-morbid patients (identified by polypharmacy) are more likely to be prescribed an antipsychotic drug, although is likely that the behaviours that would merit the prescription of an anti-psychotic tend to occur in the later stages of the illness and therefore the patient may be receiving prescriptions for other conditions. Another possibility is that multi-morbid patients may be more likely to live in residential care facilities where prescribing of antipsychotic drugs has remained high despite the National Dementia Strategy [35]. The significantly higher prescribing in Wales and Northern Ireland may be selection bias resulting from the small number of CPRD practices from these countries.

### Strengths and Limitations of the Study

In this large comprehensive study of antipsychotic drug prescribing to patients with dementia, we have considered ‘end of life’ prescribing by excluding patients who died within the 6 months leading up to the audit date from the denominator. We also allowed for the clinically appropriate short-term prescribing as described in the NICE guidelines by only including prescribing occurring for at least 3 months in the numerator. It is possible that changes in clinicians’ recording of psychosis diagnoses could influence our results but our sensitivity analyses showed that the prevalence ratios describing changes over time were similar for patients with, or without, a recorded psychosis diagnosis.

The main limitation is that any hypothesised relationship between the implementation of guidelines or warnings and changes in prescribing is based purely on a temporal association. We have no direct evidence of causation. Other interventions besides these that we selected for formal evaluation, e.g., the formation of the Dementia Action Alliance in 2010, may have had an impact [26]. Local policies might arise in response to the preceding policy intervention, thus we should consider the changes over a longer time frame as well as the immediate impacts captured by the formal ITS analysis. Another potential complicating factor is the introduction of the Quality and Outcomes Framework, a pay-for-performance programme introduced in 2004 that rewards general practitioners for recording and managing certain chronic conditions [36]. Prevalence levels are known to increase after the introduction of a Quality and Outcomes Framework incentive, [37] and psychosis and dementia were included in the scheme at different timepoints, in 2004 (as part of severe mental illness) and 2006, respectively. Nevertheless, our results are largely unaffected by the inclusion or not of patients with psychosis, while changes in dementia prevalence over time are less pertinent to our research question.

## Conclusions and Implications for the Future

In 2014, 8.2% of older patients with a diagnosis of dementia without psychosis were prescribed a second-generation antipsychotic drug for at least 3 months. Warnings and guidelines appear to have had a marked, albeit in some cases short-lived, impact on clinical practice. A further carefully worded warning may be justified to reduce the longer-term prescribing of risperidone without precipitating an increase in the prescribing of other types of antipsychotic drugs (or sedatives or antidepressants). Given the high variation in prevalence between practices, a more targeted intervention may be required, possibly implemented through the numerous computer systems that are active in UK primary care [38] or through medication review by clinical pharmacists working in general practices as endorsed by the NICE Medicines Optimisation guidance [39]. Prescribing in primary care can occur at the request of a psychiatrist or it may be initiated by a general practitioner. Further work could examine whether prescribing to patients referred to psychiatrists differed to those without referral. Nonetheless, it is important that general practitioners and psychiatrists work together to address this problem.

In general, the impact of drug safety warnings and guidelines will depend on multiple factors, [40] yet these factors are rarely investigated in relation to the type of intervention and its method of dissemination. Increased surveillance of the effectiveness of drug safety warnings and guidance is needed to improve the impact of future interventions.

## Electronic supplementary material

Below is the link to the electronic supplementary material.

Electronic Supplementary Material 1: List of codes to describe dementia and psychosis diagnoses and antipsychotic drugs (PDF 147 kb)

Electronic Supplementary Material 2: Description of population, sensitivity and other additional analyses (PDF 421 kb)

Electronic Supplementary Material 3: Interrupted time series plots by class and individual antipsychotic drug (PDF 197 kb)
