# Correction to: Antipsychotic Prescribing to Patients Diagnosed with Dementia Without a Diagnosis of Psychosis in the Context of National Guidance and Drug Safety Warnings: Longitudinal Study in UK General Practice

**DOI:** 10.1007/s40264-019-00817-2

**Published:** 2019-04-08

**Authors:** S. Jill Stocks, Evangelos Kontopantelis, Roger T. Webb, Anthony J. Avery, Alistair Burns, Darren M. Ashcroft

**Affiliations:** 1 Division of Population Health, Health Services Research and Primary Care, School of Health Sciences, National Institute for Health Research Greater Manchester Primary Care Patient Safety Translational Research Centre, Centre for Primary Care, University of Manchester, Manchester, UK; 2 Division of Population Health, Health Services Research and Primary Care, School of Health Sciences, National Institute for Health Research School for Primary Care Research, Centre for Primary Care, University of Manchester, Manchester, UK; 3 Division of Informatics, Imaging and Data Sciences, School of Health Sciences, Centre for Health Informatics, University of Manchester, Manchester, UK; 4 Division of Psychology and Mental Health, School of Health Sciences, University of Manchester, Manchester, UK; 5 Division of Primary Care, School of Medicine, Queen’s Medical Centre, University of Nottingham, Nottingham, UK; 6 Division of Neuroscience and Experimental Psychology, School of Biological Sciences, University of Manchester, Manchester, UK; 7 Division of Pharmacy and Optometry, School of Health Sciences, Centre for Pharmacoepidemiology and Drug Safety, University of Manchester, Manchester Academic Health Sciences Centre, Manchester, UK

## Correction to: Drug Saf (2017) 40:679–692 10.1007/s40264-017-0538-x

The Open Access license, which previously read:

**Open Access** This article is distributed under the terms of the Creative Commons Attribution-NonCommercial 4.0 International License (http://creativecommons.org/licenses/by-nc/4.0/), which permits any noncommercial use, distribution, and reproduction in any medium, provided you give appropriate credit to the original author(s) and the source, provide a link to the Creative Commons license, and indicate if changes were made.

Should read:

**Open Access** This article is distributed under the terms of the Creative Commons Attribution 4.0 International License (http://creativecommons.org/licenses/by/4.0/), which permits unrestricted use, distribution, and reproduction in any medium, provided you give appropriate credit to the original author(s) and the source, provide a link to the Creative Commons license, and indicate if changes were made.

The original article has been corrected.

