# Supplementary material for: Antipsychotic Prescribing to Patients Diagnosed with Dementia Without a Diagnosis of Psychosis in the Context of National Guidance and Drug Safety Warnings: Longitudinal Study in UK General Practice
Source: Drug Saf. 2017 Apr 24;40(8):679–92. doi: 10.1007/s40264-017-0538-x (PMC5519656; doi:10.1007/s40264-017-0538-x)
Supplement: Supplementary file 1 — Electronic Supplementary Material 1: List of codes to describe dementia and psychosis diagnoses and antipsychotic drugs (PDF 147 kb) [file 40264_2017_538_MOESM1_ESM.pdf]

Supplementary Appendix 1.

Antipsychotic prescribing to patients diagnosed with dementia in the context of national guidance and drug safety warnings: longitudinal study in UK general practice

S Jill Stocks, Evangelos Kontopantelis, Roger T Webb, Anthony J Avery, Alistair Burns, Darren M Ashcroft

Correspondence to Dr Jill Stocks; NIHR Greater Manchester Primary Care Patient Safety Translational Research Centre, Centre for Primary Care, Division of Population Health, Health Services Research and Primary Care, School of Health Sciences, University of Manchester, Manchester M13 9PL, UK
jill.stocks@manchester.ac.uk

Table A. Dementia diagnosis codes

Read code	Description	medcode
A411.00	Jakob-Creutzfeldt disease	38286
E00..00	Senile and presenile organic psychotic conditions	33707
E00..11	Senile dementia	1916
E00..12	Senile/presenile dementia	1350
E000.00	Uncomplicated senile dementia	7323
E001.00	Presenile dementia	15165
E001000	Uncomplicated presenile dementia	42602
E001100	Presenile dementia with delirium	49513
E001200	Presenile dementia with paranoia	30032
E001300	Presenile dementia with depression	27677
E001z00	Presenile dementia NOS	38438
E002.00	Senile dementia with depressive or paranoid features	44674
E002000	Senile dementia with paranoia	18386
E002100	Senile dementia with depression	21887
E002z00	Senile dementia with depressive or paranoid features NOS	41089
E003.00	Senile dementia with delirium	37015
E004.00	Arteriosclerotic dementia	19477
E004.11	Multi infarct dementia	8634
E004000	Uncomplicated arteriosclerotic dementia	43089
E004100	Arteriosclerotic dementia with delirium	56912
E004200	Arteriosclerotic dementia with paranoia	55467
E004300	Arteriosclerotic dementia with depression	43292
E004z00	Arteriosclerotic dementia NOS	42279
E00y.00	Other senile and presenile organic psychoses	15249
E00y.11	Presbyophrenic psychosis	51494
E00z.00	Senile or presenile psychoses NOS	2882
E012.00	Other alcoholic dementia	54505
E012.11	Alcoholic dementia NOS	27342
E012000	Chronic alcoholic brain syndrome	37946
E02y100	Drug-induced dementia	62132
E041.00	Dementia in conditions EC	25386
Eu00.00	[X]Dementia in Alzheimer's disease	7664

Eu00000	[X]Dementia in Alzheimer's disease with early onset	49263
Eu00011	[X]Presenile dementia,Alzheimer's type	25704
Eu00012	[X]Primary degen dementia, Alzheimer's type, presenile onset	60059
Eu00013	[X]Alzheimer's disease type 2	61528
Eu00100	[X]Dementia in Alzheimer's disease with late onset	38678
Eu00111	[X]Alzheimer's disease type 1	46762
Eu00112	[X]Senile dementia,Alzheimer's type	11379
Eu00113	[X]Primary degen dementia of Alzheimer's type, senile onset	43346
Eu00200	[X]Dementia in Alzheimer's dis, atypical or mixed type	30706
Eu00z00	[X]Dementia in Alzheimer's disease, unspecified	29386
Eu00z11	[X]Alzheimer's dementia unspec	8195
Eu01.00	[X]Vascular dementia	6578
Eu01.11	[X]Arteriosclerotic dementia	9565
Eu01000	[X]Vascular dementia of acute onset	46488
Eu01100	[X]Multi-infarct dementia	11175
Eu01111	[X]Predominantly cortical dementia	55838
Eu01200	[X]Subcortical vascular dementia	8934
Eu01300	[X]Mixed cortical and subcortical vascular dementia	31016
Eu01y00	[X]Other vascular dementia	55313
Eu01z00	[X]Vascular dementia, unspecified	19393
Eu02.00	[X]Dementia in other diseases classified elsewhere	12621
Eu02000	[X]Dementia in Pick's disease	28402
Eu02100	[X]Dementia in Creutzfeldt-Jakob disease	54106
Eu02200	[X]Dementia in Huntington's disease	37014
Eu02300	[X]Dementia in Parkinson's disease	9509
Eu02400	[X]Dementia in human immunodef virus [HIV] disease	41185
Eu02500	[X]Lewy body dementia	26270
Eu02y00	[X]Dementia in other specified diseases classif elsewhere	64267
Eu02z00	[X] Unspecified dementia	4693
Eu02z11	[X] Presenile dementia NOS	48501
Eu02z12	[X] Presenile psychosis NOS	47619
Eu02z13	[X] Primary degenerative dementia NOS	34944
Eu02z14	[X] Senile dementia NOS	4357
Eu02z15	[X] Senile psychosis NOS	27935
Eu02z16	[X] Senile dementia, depressed or paranoid type	27759
Eu04100	[X]Delirium superimposed on dementia	53446
Eu05700	[X]Mild cognitive disorder	11936
F110.00	Alzheimer's disease	1917
F110000	Alzheimer's disease with early onset	16797
F110100	Alzheimer's disease with late onset	32057
F111.00	Pick's disease	11136
F1110A		89580
F112.00	Senile degeneration of brain	29512
F116.00	Lewy body disease	7572
F118.00		104534
F11x.00	Cerebral degeneration in other disease EC	97422

F11x000	Cerebral degeneration due to alcoholism	47555
F11x011	Alcoholic encephalopathy	36748
F11x200	Cerebral degeneration due to cerebrovascular disease	54744
F11x400	Cerebral degeneration due to neoplastic disease	70957
F11x500	Cerebral degeneration due to myxoedema	47658
F11x600	Cerebral degeneration due to vitamin B12 deficiency	24581
F11x700	Cerebral degeneration due to Jakob - Creutzfeldt disease	48531
F11x800	Cerebral degeneration due to multifocal leucoencephalopathy	99684
F11x900	Cerebral degeneration in Parkinson's disease	96860
F11xz00	Cerebral degeneration other disease NOS	44592
F11y.00	Other cerebral degeneration	34976
F11y000	Reye's syndrome	33353
F11y100	Cerebral ataxia	15261
F11yz00	Other cerebral degeneration NOS	31524
F11z.00	Cerebral degeneration NOS	5651
F11z.11	Cerebral atrophy	2731
F134.00	Huntington's chorea	3591
Fyu3000	[X]Other Alzheimer's disease	59122

Table B. Psychosis diagnosis codes

Read code	Description	medcode
E001200	Presenile dementia with paranoia	30032
E002000	Senile dementia with paranoia	18386
E002z00	Senile dementia with depressive or paranoid features NOS	41089
E004200	Arteriosclerotic dementia with paranoia	55467
E00y.00	Other senile and presenile organic psychoses	15249
E00y.11	Presbyophrenic psychosis	51494
E00z.00	Senile or presenile psychoses NOS	2882
E04..00	Other chronic organic psychoses	62087
E04y.00	Other specified chronic organic psychoses	23677
E04z.00	Chronic organic psychosis NOS	39625
E0y..00	Other specified organic psychoses	66545
E0z..00	Organic psychoses NOS	36861
E10..00	Schizophrenic disorders	854
E100.00	Simple schizophrenia	32222
E100.11	Schizophrenia simplex	73295
E100000	Unspecified schizophrenia	15733
E100100	Subchronic schizophrenia	23616
E100200	Chronic schizophrenic	3984
E100300	Acute exacerbation of subchronic schizophrenia	57666
E100400	Acute exacerbation of chronic schizophrenia	44498
E100500	Schizophrenia in remission	58687
E100z00	Simple schizophrenia NOS	53625
E101.00	Hebephrenic schizophrenia	30619
E101000	Unspecified hebephrenic schizophrenia	66506

E101400	Acute exacerbation of chronic hebephrenic schizophrenia	97919
E101500	Hebephrenic schizophrenia in remission	67768
E101z00	Hebephrenic schizophrenia NOS	48054
E102.00	Catatonic schizophrenia	25546
E102000	Unspecified catatonic schizophrenia	58716
E102100	Subchronic catatonic schizophrenia	99199
E102500	Catatonic schizophrenia in remission	102427
E102z00	Catatonic schizophrenia NOS	63867
E103.00	Paranoid schizophrenia	1494
E103000	Unspecified paranoid schizophrenia	33383
E103200	Chronic paranoid schizophrenia	31362
E103300	Acute exacerbation of subchronic paranoid schizophrenia	51322
E103400	Acute exacerbation of chronic paranoid schizophrenia	53032
E103500	Paranoid schizophrenia in remission	36172
E103z00	Paranoid schizophrenia NOS	9281
E104.00	Acute schizophrenic episode	576
E104.11	Oneirophrenia	93167
E105.00	Latent schizophrenia	66410
E105000	Unspecified latent schizophrenia	102311
E105200	Chronic latent schizophrenia	94299
E105500	Latent schizophrenia in remission	96883
E105z00	Latent schizophrenia NOS	102446
E106.00	Residual schizophrenia	38063
E107.00	Schizo-affective schizophrenia	2117
E107.11	Cyclic schizophrenia	99000
E107000	Unspecified schizo-affective schizophrenia	58862
E107100	Subchronic schizo-affective schizophrenia	61098
E107200	Chronic schizo-affective schizophrenia	43800
E107300	Acute exacerbation subchronic schizo-affective schizophrenia	58866
E107400	Acute exacerbation of chronic schizo-affective schizophrenia	63478
E107500	Schizo-affective schizophrenia in remission	56438
E107z00	Schizo-affective schizophrenia NOS	10575
E10y.00	Other schizophrenia	39062
E10y.11	Cenesthopathic schizophrenia	92994
E10y000	Atypical schizophrenia	33338
E10y100	Coenesthopathic schizophrenia	99070
E10yz00	Other schizophrenia NOS	49761
E10z.00	Schizophrenia NOS	8407
E111.00	Recurrent manic episodes	26227
E111000	Recurrent manic episodes, unspecified	19967
E111100	Recurrent manic episodes, mild	46425
E111200	Recurrent manic episodes, moderate	27739
E111300	Recurrent manic episodes, severe without mention psychosis	65811
E111400	Recurrent manic episodes, severe, with psychosis	32295
E111500	Recurrent manic episodes, partial or unspecified remission	58863
E111600	Recurrent manic episodes, in full remission	37178

E111z00	Recurrent manic episode NOS	46415
E113400	Recurrent major depressive episodes, severe, with psychosis	24171
E114.00	Bipolar affective disorder, currently manic	3702
E114.11	Manic-depressive - now manic	17385
E114000	Bipolar affective disorder, currently manic, unspecified	35738
E114100	Bipolar affective disorder, currently manic, mild	36126
E114200	Bipolar affective disorder, currently manic, moderate	46434
E114300	Bipolar affect disord, currently manic, severe, no psychosis	16347
E114400	Bipolar affect disord, currently manic,severe with psychosis	55829
E114500	Bipolar affect disord,currently manic, part/unspec remission	59011
E114600	Bipolar affective disorder, currently manic, full remission	63784
E114z00	Bipolar affective disorder, currently manic, NOS	57605
E115.00	Bipolar affective disorder, currently depressed	4677
E115.11	Manic-depressive - now depressed	12831
E115000	Bipolar affective disorder, currently depressed, unspecified	15923
E115100	Bipolar affective disorder, currently depressed, mild	35734
E115200	Bipolar affective disorder, currently depressed, moderate	27890
E115300	Bipolar affect disord, now depressed, severe, no psychosis	35607
E115400	Bipolar affect disord, now depressed, severe with psychosis	63701
E115500	Bipolar affect disord, now depressed, part/unspec remission	72026
E115600	Bipolar affective disorder, now depressed, in full remission	57465
E115z00	Bipolar affective disorder, currently depressed, NOS	37296
E116.00	Mixed bipolar affective disorder	31316
E116000	Mixed bipolar affective disorder, unspecified	31535
E116100	Mixed bipolar affective disorder, mild	24689
E116200	Mixed bipolar affective disorder, moderate	63150
E116300	Mixed bipolar affective disorder, severe, without psychosis	63284
E116400	Mixed bipolar affective disorder, severe, with psychosis	54195
E116500	Mixed bipolar affective disorder, partial/unspec remission	63651
E116600	Mixed bipolar affective disorder, in full remission	55064
E116z00	Mixed bipolar affective disorder, NOS	63583
E117.00	Unspecified bipolar affective disorder	14784
E117000	Unspecified bipolar affective disorder, unspecified	49763
E117100	Unspecified bipolar affective disorder, mild	63698
E117200	Unspecified bipolar affective disorder, moderate	68647
E117300	Unspecified bipolar affective disorder, severe, no psychosis	73423
E117400	Unspecified bipolar affective disorder,severe with psychosis	68326
E117500	Unspecified bipolar affect disord, partial/unspec remission	70721
E117600	Unspecified bipolar affective disorder, in full remission	24230
E117z00	Unspecified bipolar affective disorder, NOS	27986
E11y.00	Other and unspecified manic-depressive psychoses	60178
E11y000	Unspecified manic-depressive psychoses	11596
E11y100	Atypical manic disorder	70925
E11y200	Atypical depressive disorder	27491
E11y300	Other mixed manic-depressive psychoses	70399
E11yz00	Other and unspecified manic-depressive psychoses NOS	33426

E11z.00	Other and unspecified affective psychoses	41992
E11z000	Unspecified affective psychoses NOS	54607
E11zz00	Other affective psychosis NOS	33425
E12..00	Paranoid states	4261
E120.00	Simple paranoid state	14743
E121.00	Chronic paranoid psychosis	3890
E122.00	Paraphrenia	14971
E123.00	Shared paranoid disorder	62680
E123.11	Folie a deux	50868
E12y.00	Other paranoid states	31589
E12y000	Paranoia querulans	66766
E12yz00	Other paranoid states NOS	31455
E12z.00	Paranoid psychosis NOS	12771
E13..00	Other nonorganic psychoses	31984
E130.00	Reactive depressive psychosis	8478
E130.11	Psychotic reactive depression	17770
E134.00	Psychogenic paranoid psychosis	24345
E141.00	Disintegrative psychosis	56143
E141.11	Heller's syndrome	31599
E141100	Residual disintegrative psychoses	41207
E14y.00	Other childhood psychoses	71819
E14y000	Atypical childhood psychoses	24244
E14y100	Borderline psychosis of childhood	69155
E14yz00	Other childhood psychoses NOS	66757
E14z.00	Child psychosis NOS	52849
E14z.11	Childhood schizophrenia NOS	37395
E1y..00	Other specified non-organic psychoses	16537
E1z..00	Non-organic psychosis NOS	22188
Eu05000	[X]Organic hallucinosis	25338
Eu05200	[X]Organic delusional [schizophrenia-like] disorder	21986
Eu10700	[X]Men & behav dis due alcoh: resid & late-onset psychot dis	62000
Eu11700	[X]Men & beh dis due opioids: resid & late-onset psychot dis	27652
Eu12700	[X]Mnt/bh dis due cannabinds: resid & late-onset psychot dis	57574
Eu14700	[X]Men & beh dis due cocaine: resid & late-onset psychot dis	102591
Eu15700	[X]Mnt/bh dis oth stm inc caffne resid/late-onset psycht dis	59163
Eu16711	[X]Post hallucinogen perception disorder	35196
Eu18500	[X]Mental & behav dis due to vol solvents: psychotic disordr	98618
Eu16500	[X]Mental & behav dis due to hallucinogens: psychotic disord	54983
Eu19700	[X]Men/beh dis mlt drg use/oth subs: resid/late psychot dis	56948
Eu20.00	[X]Schizophrenia	34236
Eu20000	[X]Paranoid schizophrenia	16764
Eu20011	[X]Paraphrenic schizophrenia	50060
Eu20100	[X]Hebephrenic schizophrenia	43405
Eu20111	[X]Disorganised schizophrenia	53985
Eu20200	[X]Catatonic schizophrenia	61501
Eu20211	[X]Catatonic stupor	20572

Eu20212	[X]Schizophrenic catalepsy	64533
Eu20213	[X]Schizophrenic catatonia	35877
Eu20214	[X]Schizophrenic flexibilatis cerea	31493
Eu20300	[X]Undifferentiated schizophrenia	60013
Eu20311	[X]Atypical schizophrenia	91547
Eu20400	[X]Post-schizophrenic depression	20785
Eu20500	[X]Residual schizophrenia	64264
Eu20511	[X]Chronic undifferentiated schizophrenia	24107
Eu20600	[X]Simple schizophrenia	35848
Eu20y00	[X]Other schizophrenia	49420
Eu20y12	[X]Schizophreniform disord NOS	94001
Eu20y13	[X]Schizophrenifrm psychos NOS	18053
Eu20z00	[X]Schizophrenia, unspecified	34966
1BH..00	Delusions	1915
1BH..11	Delusion	17982
1BH0.00	Delusion of persecution	43462
1BH1.00	Grandiose delusions	32875
1BH2.00	Ideas of reference	55479
1BH3.00	Paranoid ideation	22643
E001100	Presenile dementia with delirium	49513
E003.00	Senile dementia with delirium	37015
E004100	Arteriosclerotic dementia with delirium	56912
E010.00	Alcohol withdrawal delirium	16225
E010.11	DTs - delirium tremens	22277
E010.12	Delirium tremens	1476
E011000	Korsakov's alcoholic psychosis	4500
E013.00	Alcohol withdrawal hallucinosis	25110
E015.00	Alcoholic paranoia	30404
E01yz00	Other alcoholic psychosis NOS	68111
E01z.00	Alcoholic psychosis NOS	67651
E021.00	Drug-induced paranoia or hallucinatory states	45997
E021000	Drug-induced paranoid state	12628
E021100	Drug-induced hallucinosis	20026
E021z00	Drug-induced paranoia or hallucinatory state NOS	26481
E02y000	Drug-induced delirium	29783
E02yz00	Other drug psychoses NOS	28767
E02z.00	Drug psychosis NOS	26002
E03..00	Transient organic psychoses	55150
E030.00	Acute confusional state	4033
E030.11	Delirium - acute organic	22466
E030.12	Toxic confusional state	3486
E030000	Acute confusional state, post traumatic	25051
E030100	Acute confusional state, of infective origin	50683
E030200	Acute confusional state, of endocrine origin	94079
E030300	Acute confusional state, of metabolic origin	70409
E030400	Acute confusional state, of cerebrovascular origin	25114

E030z00	Acute confusional state NOS	41537
E031.00	Subacute confusional state	17021
E031.11	Delirium - subacute organic	24077
E031000	Subacute confusional state, post traumatic	61238
E031100	Subacute confusional state, of infective origin	69359
E031300	Subacute confusional state, of metabolic origin	38671
E031400	Subacute confusional state, of cerebrovascular origin	24035
E031z00	Subacute confusional state NOS	55784
E03y.00	Other transient organic psychoses	68201
E03y000	Organic delusional syndrome	10543
E03y100	Organic hallucinosis syndrome	47109
E03y200	Organic affective syndrome	24510
E03y300	Unspecified puerperal psychosis	2114
E03yz00	Other transient organic psychoses NOS	59197
E03z.00	Transient organic psychoses NOS	57637
E110.00	Manic disorder, single episode	37070
E110.11	Hypomanic psychoses	18909
E110000	Single manic episode, unspecified	20110
E110100	Single manic episode, mild	14728
E110200	Single manic episode, moderate	24640
E110300	Single manic episode, severe without mention of psychosis	43093
E110400	Single manic episode, severe, with psychosis	50218
E110600	Single manic episode in full remission	70000
E110z00	Manic disorder, single episode NOS	36611
E112400	Single major depressive episode, severe, with psychosis	32159
E131.00	Acute hysterical psychosis	29937
E133.00	Acute paranoid reaction	15053
E13y.00	Other reactive psychoses	16333
E13y000	Psychogenic stupor	22117
E13y100	Brief reactive psychosis	23538
E13yz00	Other reactive psychoses NOS	26119
Eu04.00	[X]Delirium, not induced by alcohol+other psychoactive subs	25066
Eu04.11	[X]Acute / subacute brain syndrome	39337
Eu04.12	[X]Acute / subacute confusional state, nonalcoholic	7389
Eu04.13	[X]Acute / subacute infective psychosis	24387
Eu04.14	[X]Acute / subacute organic reaction	21933
Eu04.15	[X]Acute / subacute psycho-organic reaction	22065
Eu04000	[X]Delirium not superimposed on dementia, so described	68125
Eu04100	[X]Delirium superimposed on dementia	53446
Eu04y00	[X]Other delirium	52394
Eu04z00	[X]Delirium, unspecified	53924
Eu10400	[X]Men & behav dis due alcohl: withdrawl state with delirium	64101
Eu10411	[X]Delirium tremens, alcohol induced	17259
Eu10500	[X]Mental & behav dis due to use alcohol: psychotic disorder	12353
Eu10511	[X]Alcoholic hallucinosis	6467
Eu10512	[X]Alcoholic jealousy	65932

Eu10513	[X]Alcoholic paranoia	30162
Eu10514	[X]Alcoholic psychosis NOS	17607
Eu11400	[X]Men & behav dis due opioid: withdrawl state with delirium	97488
Eu11500	[X]Mental & behav dis due to use opioids: psychotic disorder	50964
Eu12500	[X]Mental & behav dis due to cannabinoids: psychotic disordr	38429
Eu13400	[X]Men & beh dis due seds/hypns: withdrwl state wth delirium	44131
Eu13500	[X]Mental & behav dis due to seds/hypntcs: psychotic disordr	69138
Eu14500	[X]Mental & behav dis due to use cocaine: psychotic disorder	49565
Eu15500	[X]Mental/behav dis oth stims inc caffeine: psychotic dis	49879
Eu16500	[X]Mental & behav dis due to hallucinogens: psychotic disord	54983
Eu18400	[X]Men & beh dis vol solvents: withdrawal state wth delirium	62106
Eu18500	[X]Mental & behav dis due to vol solvents: psychotic disordr	98618
Eu19400	[X]Mnt/bh dis mlti drg use/oth psy sbs: wthdr state + dlrium	65950
Eu19500	[X]Ment/behav dis mlti drug use/oth psyc sbs: psychotc dis	24637
Eu1A500	[X]Mental behav disord due crack cocaine: psychotic disorder	102475
Eu23.00	[X]Acute and transient psychotic disorders	25019
Eu23000	[X]Acute polymorphic psychot disord without symp of schizoph	36720
Eu23011	[X]Bouffee delirante	50023
Eu23012	[X]Cycloid psychosis	21455
Eu23100	[X]Acute polymorphic psychot disord with symp of schizophren	21595
Eu23112	[X]Cycloid psychosis with symptoms of schizophrenia	26143
Eu23200	[X]Acute schizophrenia-like psychotic disorder	11778
Eu23211	[X]Brief schizophreniform disorder	59096
Eu23212	[X]Brief schizophrenifrm psych	70884
Eu23214	[X]Schizophrenic reaction	94604
Eu23300	[X]Other acute predominantly delusional psychotic disorders	44307
Eu23312	[X]Psychogenic paranoid psychosis	27770
Eu23y00	[X]Other acute and transient psychotic disorders	44503
Eu23z00	[X]Acute and transient psychotic disorder, unspecified	34168
Eu23z11	[X]Brief reactive psychosis NOS	31707
Eu23z12	[X]Reactive psychosis	29651
Eu24.00	[X]Induced delusional disorder	51302
Eu24.12	[X]Induced paranoid disorder	47230
Eu24.13	[X]Induced psychotic disorder	11973
R001.00	[D]Hallucinations	2455
R001000	[D]Hallucinations, auditory	12120
R001100	[D]Hallucinations, gustatory	53990
R001200	[D]Hallucinations, olfactory	25283
R001300	[D]Hallucinations, tactile	64131
R001400	[D]Visual hallucinations	12064
R001z00	[D]Hallucinations NOS	19916

Table C. Antipsychotic drug codes

prodcode	Description	Drug substance
4876	Amisulpride 50mg tablets	Amisulpride
4992	Solian 200 tablets (Sanofi)	Amisulpride
5071	Amisulpride 200mg tablets	Amisulpride
5927	Amisulpride 400mg tablets	Amisulpride
6109	Solian 400 tablets (Sanofi)	Amisulpride
6482	Amisulpride 100mg/ml oral solution sugar free	Amisulpride
6524	Amisulpride 100mg tablets	Amisulpride
11938	Amisulpride 25mg/5ml oral suspension	Amisulpride
16768	Solian 50 tablets (Sanofi)	Amisulpride
26544	Solian 100 tablets (Sanofi)	Amisulpride
31576	Solian 100mg/ml oral solution (Sanofi)	Amisulpride
34927	Amisulpride 200mg tablets (Zentiva)	Amisulpride
41702	Amisulpride 100mg tablets (Zentiva)	Amisulpride
41714	Amisulpride 50mg tablets (Zentiva)	Amisulpride
46889	Amisulpride 25mg/5ml oral solution	Amisulpride
46969	Amisulpride 200mg tablets (A A H Pharmaceuticals Ltd)	Amisulpride
55625	Amisulpride 50mg/5ml oral suspension	Amisulpride
6894	Perphenazine 2mg with Amitriptyline 25mg tablet	Amitriptyline Hydrochloride
16323	Perphenazine 2mg with Amitriptyline 10mg tablet	Amitriptyline Hydrochloride
1453	Triptafen m 2mg+10mg Tablet (Goldshield Pharmaceuticals Ltd)	Amitriptyline hydrochloride/Perphenazine
6561	Aripiprazole 10mg tablets	Aripiprazole
6573	Aripiprazole 15mg tablets	Aripiprazole
14344	Aripiprazole 5mg tablets	Aripiprazole
14858	Abilify 15mg tablets (Otsuka Pharmaceuticals (U.K.) Ltd)	Aripiprazole
16561	Aripiprazole 30mg tablets	Aripiprazole
16575	Aripiprazole 1mg/ml oral solution	Aripiprazole
18132	Abilify 5mg tablets (Otsuka Pharmaceuticals (U.K.) Ltd)	Aripiprazole
24358	Abilify 10mg tablets (Otsuka Pharmaceuticals (U.K.) Ltd)	Aripiprazole
29879	Abilify 30mg tablets (Otsuka Pharmaceuticals (U.K.) Ltd)	Aripiprazole
31098	Aripiprazole 15mg orodispersible tablets sugar free	Aripiprazole
32076	Aripiprazole 10mg orodispersible tablets sugar free	Aripiprazole
37606	Abilify 10mg orodispersible tablets (Otsuka Pharmaceuticals (U.K.) Ltd)	Aripiprazole
38010	Abilify 15mg orodispersible tablets (Otsuka Pharmaceuticals (U.K.) Ltd)	Aripiprazole
38080	Abilify 1mg/ml oral solution (Otsuka Pharmaceuticals (U.K.) Ltd)	Aripiprazole
38375	Aripiprazole 9.75mg/1.3ml solution for injection vials	Aripiprazole
46705	Abilify 9.75mg/1.3ml solution for injection vials (Otsuka Pharmaceuticals (U.K.) Ltd)	Aripiprazole

49699	Abilify 5mg tablets (Sigma Pharmaceuticals Plc)	Aripiprazole
57114	Abilify 5mg tablets (Mawdsley-Brooks & Company Ltd)	Aripiprazole
47167	Asenapine 10mg sublingual tablets sugar free	Asenapine
47280	Asenapine 5mg sublingual tablets sugar free	Asenapine
2540	Benperidol 250microgram tablets	Benperidol
21744	Anquil 250microgram Tablet (Concord Pharmaceuticals Ltd)	Benperidol
31796	Benquil 250microgram tablets (Concord Pharmaceuticals Ltd)	Benperidol
47365	Anquil 250microgram tablets (Archimedes Pharma UK Ltd)	Benperidol
15418	Largactil forte 100mg/5ml Oral suspension (Hawgreen Ltd)	Chlorpromazine embonate
588	Chlorpromazine 25mg tablets	Chlorpromazine hydrochloride
2154	Chlorpromazine 100mg tablets	Chlorpromazine hydrochloride
2814	Largactil 25mg Tablet (Hawgreen Ltd)	Chlorpromazine hydrochloride
3348	Chlorpromazine 50mg tablets	Chlorpromazine hydrochloride
3772	Largactil 50mg Tablet (Hawgreen Ltd)	Chlorpromazine hydrochloride
3952	Chlorpromazine 25mg/5ml oral solution	Chlorpromazine hydrochloride
4434	Chlorpromazine 50mg/5ml oral solution	Chlorpromazine hydrochloride
7493	Largactil 100mg Tablet (Hawgreen Ltd)	Chlorpromazine hydrochloride
7514	Largactil 50mg/2ml solution for injection ampoules (Sanofi)	Chlorpromazine hydrochloride
8311	Chlorpromazine 25mg/ml injection	Chlorpromazine Hydrochloride
8506	Chlorpromazine 100mg suppository	Chlorpromazine Hydrochloride
8519	Chlorpromazine 100mg/5ml oral solution	Chlorpromazine hydrochloride
8771	Largactil 10mg Tablet (Hawgreen Ltd)	Chlorpromazine hydrochloride
9190	Chlorpromazine 25mg/5ml oral solution sugar free	Chlorpromazine hydrochloride
10434	Largactil 25mg/5ml Oral solution (Hawgreen Ltd)	Chlorpromazine hydrochloride
17227	Chloractil 25mg Tablet (DDSA Pharmaceuticals Ltd)	Chlorpromazine hydrochloride
19002	Largactil 100mg Suppository (Rhone-Poulenc Rorer Ltd)	Chlorpromazine Hydrochloride
22606	Chlorpromazine 25mg/1ml solution for injection ampoules	Chlorpromazine hydrochloride
25653	Chloractil 50mg Tablet (DDSA Pharmaceuticals Ltd)	Chlorpromazine hydrochloride
28862	Chloractil 100mg Tablet (DDSA Pharmaceuticals Ltd)	Chlorpromazine

		hydrochloride
31171	Chlorpromazine 50mg tablets (A A H Pharmaceuticals Ltd)	Chlorpromazine hydrochloride
31172	Chlorpromazine 50mg tablets (Teva UK Ltd)	Chlorpromazine hydrochloride
31175	Chlorpromazine 25mg tablets (A A H Pharmaceuticals Ltd)	Chlorpromazine hydrochloride
31184	Chlorpromazine 25mg tablets (IVAX Pharmaceuticals UK Ltd)	Chlorpromazine hydrochloride
34630	Chlorpromazine 50mg tablets (Thornton & Ross Ltd)	Chlorpromazine hydrochloride
34668	Chlorpromazine 25mg tablets (Teva UK Ltd)	Chlorpromazine hydrochloride
34693	Chlorpromazine 25mg tablets (Thornton & Ross Ltd)	Chlorpromazine hydrochloride
34736	Chlorpromazine 100mg tablets (Teva UK Ltd)	Chlorpromazine hydrochloride
35929	Chlorpromazine 50mg/2ml solution for injection ampoules	Chlorpromazine hydrochloride
37705	Chlorpromazine 100mg/5ml suspension	Chlorpromazine Hydrochloride
37871	Chlorpromazine 25mg/5ml Oral solution (Rosemont Pharmaceuticals Ltd)	Chlorpromazine hydrochloride
41645	Chlorpromazine 25mg/ml Injection (Antigen Pharmaceuticals)	Chlorpromazine Hydrochloride
44186	Chlorpromazine 25mg/5ml oral solution (A A H Pharmaceuticals Ltd)	Chlorpromazine hydrochloride
45281	Chlorpromazine 100mg/5ml oral solution (Rosemont Pharmaceuticals Ltd)	Chlorpromazine hydrochloride
46960	Chlorpromazine 100mg tablets (IVAX Pharmaceuticals UK Ltd)	Chlorpromazine hydrochloride
55011	Largactil 25mg tablets (Sanofi)	Chlorpromazine hydrochloride
56862	Chlorpromazine 25mg/5ml syrup (Rosemont Pharmaceuticals Ltd)	Chlorpromazine hydrochloride
57550	Largactil 25mg/5ml syrup (Sanofi)	Chlorpromazine hydrochloride
58492	Chlorpromazine 100mg tablets (Waymade Healthcare Plc)	Chlorpromazine hydrochloride
58702	Largactil 100mg tablets (Sanofi)	Chlorpromazine hydrochloride
58703	Largactil 50mg tablets (Sanofi)	Chlorpromazine hydrochloride
28147	Taractan 15mg Tablet (Roche Products Ltd)	Chlorprothixene
30111	Chlorprothixene 50mg tablets	Chlorprothixene
8046	Clozapine 25mg tablets	Clozapine
8047	Clozapine 100mg tablets	Clozapine
14112	Clozaril 100mg tablets (Novartis Pharmaceuticals UK Ltd)	Clozapine
17958	Clozaril 25mg tablets (Novartis Pharmaceuticals UK Ltd)	Clozapine
21199	Denzapine 100mg tablets (Britannia Pharmaceuticals Ltd)	Clozapine

30487	Denzapine 25mg tablets (Britannia Pharmaceuticals Ltd)	Clozapine
40586	Clozapine 50mg tablets	Clozapine
40587	Clozapine 200mg tablets	Clozapine
41070	Denzapine 50mg tablets (Britannia Pharmaceuticals Ltd)	Clozapine
41428	Denzapine 200mg tablets (Britannia Pharmaceuticals Ltd)	Clozapine
42242	Clozapine 50mg/ml oral suspension sugar free	Clozapine
45444	Denzapine 50mg/ml oral suspension (Britannia Pharmaceuticals Ltd)	Clozapine
47233	Zaponex 25mg tablets (Teva UK Ltd)	Clozapine
47302	Zaponex 100mg tablets (Teva UK Ltd)	Clozapine
3773	Droperidol 10mg tablets	Droperidol
13369	Droleptan 1mg/ml Oral solution (Janssen-Cilag Ltd)	Droperidol
15128	Droperidol 1mg/ml liquid	Droperidol
15171	Droleptan 10mg Tablet (Janssen-Cilag Ltd)	Droperidol
21125	Droleptan 5mg/ml Injection (Janssen-Cilag Ltd)	Droperidol
22609	Droperidol 5mg/ml injection	Droperidol
42229	Droperidol oral liquid	Droperidol
53634	Droperidol capsules	Droperidol
1733	Flupentixol decanoate 20mg/ml Injection	Flupentixol Decanoate
2136	Depixol 20mg/ml Injection (Lundbeck Ltd)	Flupentixol Decanoate
2155	Depixol -conc 100mg/ml Injection (Lundbeck Ltd)	Flupentixol Decanoate
2156	Depixol 40mg/2ml solution for injection ampoules (Lundbeck Ltd)	Flupentixol decanoate
2276	Flupentixol 40mg/2ml solution for injection ampoules	Flupentixol decanoate
8712	Flupentixol decanoate 100mg/ml Injection	Flupentixol Decanoate
14130	Depixol Low Volume 200mg/1ml solution for injection ampoules (Lundbeck Ltd)	Flupentixol decanoate
14839	Flupentixol 200mg/1ml solution for injection ampoules	Flupentixol decanoate
14889	Depixol Conc 100mg/1ml solution for injection ampoules (Lundbeck Ltd)	Flupentixol decanoate
14966	Flupentixol 20mg/1ml solution for injection ampoules	Flupentixol decanoate
18155	Flupentixol 50mg/0.5ml solution for injection ampoules	Flupentixol decanoate
18175	Flupentixol 100mg/1ml solution for injection ampoules	Flupentixol decanoate
18197	Depixol Conc 50mg/0.5ml solution for injection ampoules (Lundbeck Ltd)	Flupentixol decanoate
19283	Depixol 20mg/1ml solution for injection ampoules (Lundbeck Ltd)	Flupentixol decanoate
57170	Psytixol 100mg/1ml solution for injection ampoules (Generics (UK) Ltd)	Flupentixol decanoate
57762	Psytixol 40mg/2ml solution for injection ampoules (Generics (UK) Ltd)	Flupentixol decanoate
59816	Psytixol 50mg/0.5ml solution for injection ampoules (Generics (UK) Ltd)	Flupentixol decanoate
5707	Flupentixol 3mg tablets	Flupentixol dihydrochloride
5712	Depixol 3mg tablets (Lundbeck Ltd)	Flupentixol dihydrochloride
55620	Flupentixol Liquid	Flupentixol

		Dihydrochloride
3926	Modecate 25mg/ml Injection (Sanofi-Synthelabo Ltd)	Fluphenazine Decanoate
9022	Fluphenazine decanoate 25mg/ml Injection	Fluphenazine Decanoate
10514	Fluphenazine decanoate 100mg/ml Injection	Fluphenazine Decanoate
12128	Modecate concentrate 100mg/ml Injection (Sanofi-Synthelabo Ltd)	Fluphenazine Decanoate
33780	Modecate 25mg/1ml solution for injection ampoules (Sanofi)	Fluphenazine decanoate
35065	Fluphenazine decanoate 25mg/1ml solution for injection ampoules	Fluphenazine decanoate
35122	Modecate 12.5mg/0.5ml solution for injection ampoules (Sanofi)	Fluphenazine decanoate
35176	Fluphenazine decanoate 100mg/1ml solution for injection ampoules	Fluphenazine decanoate
35391	Fluphenazine decanoate 50mg/0.5ml solution for injection ampoules	Fluphenazine decanoate
35445	Modecate 50mg/2ml solution for injection ampoules (Sanofi)	Fluphenazine decanoate
35455	Modecate Concentrate 100mg/1ml solution for injection ampoules (Sanofi)	Fluphenazine decanoate
35487	Modecate Concentrate 50mg/0.5ml solution for injection ampoules (Sanofi)	Fluphenazine decanoate
35530	Fluphenazine decanoate 12.5mg/0.5ml solution for injection ampoules	Fluphenazine decanoate
35723	Fluphenazine decanoate 50mg/2ml solution for injection ampoules	Fluphenazine decanoate
41970	Fluphenazine decanoate 25mg/1ml solution for injection ampoules (Hospira UK Ltd)	Fluphenazine decanoate
41971	Fluphenazine decanoate 25mg/ml Injection (Antigen Pharmaceuticals)	Fluphenazine decanoate
17190	Fluphenazine enanthate 25mg/ml Injection	Fluphenazine Enantate
25835	Moditen enanthate 25mg/ml Injection (Sanofi-Synthelabo Ltd)	Fluphenazine Enantate
5212	Fluphenazine 1mg tablets	Fluphenazine hydrochloride
5597	Moditen 1mg tablets (Sanofi)	Fluphenazine hydrochloride
329	Haloperidol 1.5mg tablets	Haloperidol
475	Haloperidol 10mg tablets	Haloperidol
2419	Haloperidol 500microgram capsules	Haloperidol
2620	Haloperidol 1mg/ml Oral solution	Haloperidol
2621	Haloperidol 5mg tablets	Haloperidol
3233	Haloperidol 2mg/ml sugar free Liquid	Haloperidol
4234	Haloperidol 5mg/ml Injection	Haloperidol
5192	Haloperidol 1mg/5ml sugar free Oral solution	Haloperidol
5545	Serenace 500microgram capsules (Teva UK Ltd)	Haloperidol
6134	Dozic 5mg/5ml oral solution (Rosemont Pharmaceuticals Ltd)	Haloperidol
6523	Haldol 5mg/ml Injection (Janssen-Cilag Ltd)	Haloperidol

7436	Serenace 5mg/1ml solution for injection ampoules (IVAX Pharmaceuticals UK Ltd)	Haloperidol
8153	Serenace 2mg/ml liquid (Teva UK Ltd)	Haloperidol
8979	Serenace 1.5mg tablets (Teva UK Ltd)	Haloperidol
9975	Haloperidol 1mg/ml sugar free Oral solution	Haloperidol
10435	Haloperidol 10mg/ml Oral solution	Haloperidol
11213	Haloperidol 2mg/5ml sugar free Oral solution	Haloperidol
12387	Haloperidol 20mg tablets	Haloperidol
12921	Haldol 2mg/ml oral solution (Janssen-Cilag Ltd)	Haloperidol
13105	Haloperidol 2mg/ml Oral solution	Haloperidol
13338	Serenace 5mg tablets (Teva UK Ltd)	Haloperidol
13483	Serenace 20mg tablets (Teva UK Ltd)	Haloperidol
13484	Serenace 10mg tablets (Teva UK Ltd)	Haloperidol
17379	Haloperidol 1.5mg/5ml sugar free Oral solution	Haloperidol
22660	Haldol 5mg tablets (Janssen-Cilag Ltd)	Haloperidol
23678	Haldol 10mg tablets (Janssen-Cilag Ltd)	Haloperidol
24494	Haldol 10mg/ml Liquid (Janssen-Cilag Ltd)	Haloperidol
28679	Dozic 2mg/ml Oral solution (Rosemont Pharmaceuticals Ltd)	Haloperidol
32051	Haloperidol 5mg Tablet (Generics (UK) Ltd)	Haloperidol
32838	Haloperidol 1.5mg tablets (IVAX Pharmaceuticals UK Ltd)	Haloperidol
34039	Haloperidol 1mg/ml Liquid (Rosemont Pharmaceuticals Ltd)	Haloperidol
34272	Haloperidol 5mg/ml Injection (Antigen Pharmaceuticals)	Haloperidol
34339	Haloperidol 1.5mg tablets (A A H Pharmaceuticals Ltd)	Haloperidol
34903	Haloperidol 5mg tablets (IVAX Pharmaceuticals UK Ltd)	Haloperidol
36771	Haloperidol 250micrograms/5ml oral suspension	Haloperidol
38262	Haloperidol 5mg/1ml solution for injection ampoules	Haloperidol
38540	Haldol 5mg/1ml solution for injection ampoules (Janssen-Cilag Ltd)	Haloperidol
41546	Haloperidol 1mg/ml Liquid (Hillcross Pharmaceuticals Ltd)	Haloperidol
42000	Haloperidol 2mg/ml Liquid (Rosemont Pharmaceuticals Ltd)	Haloperidol
42807	Haloperidol 500microgram Tablet (Lagap)	Haloperidol
42895	Haloperidol 5mg tablets (Teva UK Ltd)	Haloperidol
43020	Haloperidol Oral solution	Haloperidol
43520	Haloperidol 1.5mg tablets (Teva UK Ltd)	Haloperidol
45810	Haloperidol 10mg/5ml oral solution sugar free	Haloperidol
45880	Haloperidol 5mg/5ml oral solution sugar free	Haloperidol
47013	Haloperidol 1mg/5ml oral suspension	Haloperidol
47149	Haloperidol 1mg/5ml oral solution	Haloperidol
47808	Haloperidol 10mg/5ml oral solution sugar free (A A H Pharmaceuticals Ltd)	Haloperidol
49207	Haloperidol 2mg/5ml oral solution	Haloperidol
53649	Haloperidol 2mg/5ml oral suspension	Haloperidol
55848	Haloperidol 5mg/1ml solution for injection ampoules (AMCo)	Haloperidol

55871	Haloperidol 2mg/ml Liquid (Hillcross Pharmaceuticals Ltd)	Haloperidol
2094	Haldol decanoate 50mg/1ml solution for injection ampoules (Janssen-Cilag Ltd)	Haloperidol decanoate
10565	Haloperidol decanoate 50mg/1ml solution for injection ampoules	Haloperidol decanoate
12386	Haldol decanoate 100mg/1ml solution for injection ampoules (Janssen-Cilag Ltd)	Haloperidol decanoate
15814	Haloperidol decanoate 100mg/1ml solution for injection ampoules	Haloperidol decanoate
8445	Stelabid Tablet (GlaxoSmithKline Consumer Healthcare)	Isopropamide Iodide
4442	Nozinan 25mg/1ml solution for injection ampoules (Sanofi)	Levomepromazine hydrochloride
6064	Levomepromazine 25mg/1ml solution for injection ampoules	Levomepromazine hydrochloride
52846	Nozinan 25mg/1ml solution for injection ampoules (Lexon (UK) Ltd)	Levomepromazine hydrochloride
59938	Levomepromazine 25mg/1ml solution for injection ampoules (Wockhardt UK Ltd)	Levomepromazine hydrochloride
4232	Nozinan 25mg tablets (Sanofi)	Levomepromazine maleate
5014	Levomepromazine 25mg tablets	Levomepromazine maleate
7390	Levomepromazine 6mg tablets	Levomepromazine maleate
21339	Veractil 25mg Tablet (Rhone-Poulenc Rorer Ltd)	Levomepromazine maleate
28231	Levinan 6mg Tablet (Link Pharmaceuticals Ltd)	Levomepromazine maleate
40782	Levomepromazine 6mg Tablet	Levomepromazine Maleate
49606	Levinan 6mg tablets (Archimedes Pharma UK Ltd)	Levomepromazine maleate
53951	Levomepromazine 6.25mg/5ml oral solution	Levomepromazine maleate
60250	Levomepromazine 3mg/5ml oral solution	Levomepromazine maleate
1249	Olanzapine 10mg tablets	Olanzapine
2656	Olanzapine 2.5mg tablets	Olanzapine
3281	Olanzapine 5mg tablets	Olanzapine
5653	Olanzapine 7.5mg tablets	Olanzapine
6023	Olanzapine 10mg Orodispersible tablet	Olanzapine
6412	Olanzapine 5mg Orodispersible tablet	Olanzapine
6838	Olanzapine 15mg Orodispersible tablet	Olanzapine
6850	Olanzapine 15mg tablets	Olanzapine
13820	Zyprexa 10mg tablets (Eli Lilly and Company Ltd)	Olanzapine
13888	Zyprexa 10mg Velotabs (Eli Lilly and Company Ltd)	Olanzapine
14717	Zyprexa 5mg Velotabs (Eli Lilly and Company Ltd)	Olanzapine
16103	Olanzapine 20mg Orodispersible tablet	Olanzapine
16407	Zyprexa 15mg Velotabs (Eli Lilly and Company Ltd)	Olanzapine

18024	Zyprexa 5mg tablets (Eli Lilly and Company Ltd)	Olanzapine
18453	Zyprexa 2.5mg tablets (Eli Lilly and Company Ltd)	Olanzapine
19976	Zyprexa 15mg tablets (Eli Lilly and Company Ltd)	Olanzapine
21964	Zyprexa 7.5mg tablets (Eli Lilly and Company Ltd)	Olanzapine
23431	Olanzapine 10mg powder for solution for injection vials	Olanzapine
29540	Olanzapine 20mg tablets	Olanzapine
33883	Zyprexa 20mg Velotabs (Eli Lilly and Company Ltd)	Olanzapine
36163	Zyprexa 20mg tablets (Eli Lilly and Company Ltd)	Olanzapine
45953	Zyprexa 10mg powder for solution for injection vials (Eli Lilly and Company Ltd)	Olanzapine
47049	Olanzapine 10mg orodispersible tablets	Olanzapine
47055	Olanzapine 5mg orodispersible tablets	Olanzapine
47063	Olanzapine 10mg orodispersible tablets sugar free	Olanzapine
47083	Olanzapine 20mg orodispersible tablets	Olanzapine
47093	Olanzapine 20mg orodispersible tablets sugar free	Olanzapine
47098	Olanzapine 5mg orodispersible tablets sugar free	Olanzapine
47103	Olanzapine 15mg orodispersible tablets sugar free	Olanzapine
47152	Olanzapine 15mg orodispersible tablets	Olanzapine
47256	Olanzapine 5mg oral lyophilisates sugar free	Olanzapine
47304	Olanzapine 10mg oral lyophilisates sugar free	Olanzapine
47394	Olanzapine 15mg oral lyophilisates sugar free	Olanzapine
47498	Olanzapine 20mg oral lyophilisates sugar free	Olanzapine
52001	Olanzapine 2.5mg tablets (Aspire Pharma Ltd)	Olanzapine
53556	Olanzapine 10mg oral lyophilisates sugar free	Olanzapine
53848	Zalasta 5mg orodispersible tablets (Consilient Health Ltd)	Olanzapine
55667	Olanzapine 15mg tablets (Actavis UK Ltd)	Olanzapine
56072	Olanzapine 20mg orodispersible tablets	Olanzapine
56265	Olanzapine 20mg oral lyophilisates sugar free	Olanzapine
57160	Olanzapine 5mg oral lyophilisates sugar free	Olanzapine
57270	Olanzapine 2.5mg/5ml oral suspension	Olanzapine
57616	Olanzapine 20mg tablets (Teva UK Ltd)	Olanzapine
58147	Olanzapine 10mg tablets (Zentiva)	Olanzapine
58854	Olanzapine 10mg tablets (Actavis UK Ltd)	Olanzapine
59143	Olanzapine 2.5mg tablets (Teva UK Ltd)	Olanzapine
43914	Olanzapine embonate 210mg powder and solvent for suspension for injection vials	Olanzapine embonate monohydrate
46422	Olanzapine embonate 300mg powder and solvent for suspension for injection vials	Olanzapine embonate monohydrate
55268	Zypadhera 300mg powder and solvent for suspension for injection vials (Eli Lilly and Company Ltd)	Olanzapine embonate monohydrate
8921	Integrin 10mg Capsule (Sanofi-Synthelabo Ltd)	Oxypertine
27211	Integrin 40mg Tablet (Sanofi-Synthelabo Ltd)	Oxypertine
36116	Paliperidone 6mg modified-release tablets	Paliperidone
36954	Invega 6mg modified-release tablets (Janssen-Cilag Ltd)	Paliperidone
37501	Paliperidone 9mg modified-release tablets	Paliperidone
37717	Paliperidone 3mg modified-release tablets	Paliperidone
46224	Paliperidone 50mg/0.5ml suspension for injection pre-	Paliperidone Palmitate

	filled syringes	
46351	Paliperidone 150mg/1.5ml suspension for injection pre-filled syringes	Paliperidone Palmitate
46434	Xeplion 100mg/1ml suspension for injection pre-filled syringes (Janssen-Cilag Ltd)	Paliperidone Palmitate
46435	Xeplion 150mg/1.5ml suspension for injection pre-filled syringes (Janssen-Cilag Ltd)	Paliperidone palmitate
46436	Xeplion 75mg/0.75ml suspension for injection pre-filled syringes (Janssen-Cilag Ltd)	Paliperidone palmitate
46447	Paliperidone 100mg/1ml suspension for injection pre-filled syringes	Paliperidone Palmitate
46556	Paliperidone 75mg/0.75ml suspension for injection pre-filled syringes	Paliperidone Palmitate
47162	Xeplion 50mg/0.5ml suspension for injection pre-filled syringes (Janssen-Cilag Ltd)	Paliperidone Palmitate
7833	Neulactil 2.5mg Tablet (JHC Healthcare Ltd)	Pericyazine
7834	Pericyazine 2.5mg tablets	Pericyazine
8031	Neulactil 10mg Tablet (JHC Healthcare Ltd)	Pericyazine
8032	Pericyazine 10mg tablets	Pericyazine
12195	Pericyazine 10mg/5ml oral solution	Pericyazine
13902	Neulactil Forte syrup (Sanofi)	Pericyazine
15472	Pericyazine 25mg tablet	Pericyazine
21064	Neulactil 25mg Tablet (JHC Healthcare Ltd)	Pericyazine
39830	Neulactil 2.5mg tablets (Sanofi)	Pericyazine
40881	Neulactil 10mg tablets (Sanofi)	Pericyazine
228	Fentazin 5mg/ml Injection (Goldshield Pharmaceuticals Ltd)	Perphenazine
609	Perphenazine 2mg tablets	Perphenazine
840	Fentazin 2mg tablets (AMCo)	Perphenazine
2157	Perphenazine 4mg tablets	Perphenazine
7919	Fentazin 4mg tablets (AMCo)	Perphenazine
14987	Perphenazine 2mg/5ml oral solution sugar free	Perphenazine
17087	Perphenazine 5mg/ml injection	Perphenazine
25909	Perphenazine 4mg/5ml Oral solution sugar free	Perphenazine
5821	Pimozide 4mg tablets	Pimozide
8637	Pimozide 10mg tablet	Pimozide
15047	Orap 4mg tablets (Janssen-Cilag Ltd)	Pimozide
27148	Orap 10mg Tablet (Janssen-Cilag Ltd)	Pimozide
10944	Pipotiazine palmitate 50mg/ml depot injection	Pipotiazine Palmitate
12340	Piportil 50mg/ml Depot injection (JHC Healthcare Ltd)	Pipotiazine Palmitate
35235	Piportil Depot 50mg/1ml solution for injection ampoules (Sanofi)	Pipotiazine palmitate
35488	Piportil Depot 100mg/2ml solution for injection ampoules (Sanofi)	Pipotiazine palmitate
35684	Pipotiazine 50mg/1ml solution for injection ampoules	Pipotiazine palmitate
36394	Pipotiazine 100mg/2ml solution for injection ampoules	Pipotiazine palmitate
2972	Promazine 25mg tablets	Promazine hydrochloride
3197	Promazine 100mg tablet	Promazine Hydrochloride

3226	Sparine 50mg Tablet (Wyeth Pharmaceuticals)	Promazine hydrochloride
3227	Sparine 50mg/5ml Liquid (Wyeth Pharmaceuticals)	Promazine Hydrochloride
3228	Promazine 50mg tablets	Promazine hydrochloride
6443	Promazine 25mg/5ml oral solution	Promazine hydrochloride
10780	Promazine 50mg/5ml oral solution	Promazine Hydrochloride
12193	Sparine 25mg Tablet (Wyeth Pharmaceuticals)	Promazine hydrochloride
13311	Sparine 50mg/ml Injection (Wyeth Pharmaceuticals)	Promazine Hydrochloride
14610	Promazine 50mg/5ml oral solution sugar free	Promazine Hydrochloride
15161	Promazine 50mg/ml injection	Promazine Hydrochloride
15395	Promazine 12.5mg/5ml oral solution	Promazine Hydrochloride
17634	Promazine 50mg/5ml oral solution	Promazine hydrochloride
33493	Sparine 100mg Tablet (Wyeth Pharmaceuticals)	Promazine Hydrochloride
38089	Promazine 50mg/5ml syrup (Rosemont Pharmaceuticals Ltd)	Promazine hydrochloride
40390	Promazine 25mg/5ml syrup (Rosemont Pharmaceuticals Ltd)	Promazine hydrochloride
41732	Promazine 50mg tablets (Teva UK Ltd)	Promazine hydrochloride
41995	Promazine 50mg/ml Injection (Genus Pharmaceuticals Ltd)	Promazine Hydrochloride
43654	Promazine 50mg/ml injection	Promazine Hydrochloride
46945	Promazine 25mg Tablet (Biorex Laboratories Ltd)	Promazine hydrochloride
55890	Promazine 50mg/5ml Liquid (Rosemont Pharmaceuticals Ltd)	Promazine Hydrochloride
60450	Promazine 25mg tablets (A A H Pharmaceuticals Ltd)	Promazine hydrochloride
5039	Quetiapine 100mg tablets	Quetiapine fumarate
5040	Quetiapine 150mg tablets	Quetiapine fumarate
5283	Quetiapine 25mg tablets	Quetiapine fumarate
6864	Seroquel 200mg tablets (AstraZeneca UK Ltd)	Quetiapine fumarate
7039	Quetiapine 300mg tablets	Quetiapine fumarate
9794	Quetiapine 200mg tablets	Quetiapine fumarate
10107	Quetiapine Starter Pack	Quetiapine Fumarate
14813	Seroquel 150mg tablets (AstraZeneca UK Ltd)	Quetiapine fumarate
14859	Seroquel 25mg tablets (AstraZeneca UK Ltd)	Quetiapine fumarate
18013	Seroquel 100mg tablets (AstraZeneca UK Ltd)	Quetiapine fumarate
21709	Seroquel 300mg tablets (AstraZeneca UK Ltd)	Quetiapine fumarate
38840	Quetiapine 400mg modified-release tablets	Quetiapine fumarate
38885	Quetiapine 50mg modified-release tablets	Quetiapine Fumarate
38906	Quetiapine 300mg modified-release tablets	Quetiapine fumarate
38912	Quetiapine 200mg modified-release tablets	Quetiapine fumarate
38913	Seroquel XL 50mg tablets (AstraZeneca UK Ltd)	Quetiapine Fumarate
38914	Seroquel XL 200mg tablets (AstraZeneca UK Ltd)	Quetiapine fumarate
38937	Seroquel XL 300mg tablets (AstraZeneca UK Ltd)	Quetiapine fumarate
39237	Seroquel XL 400mg tablets (AstraZeneca UK Ltd)	Quetiapine fumarate
40779	Quetiapine 100mg/5ml oral solution	Quetiapine fumarate
40932	Quetiapine 100mg/5ml oral suspension	Quetiapine fumarate
44024	Quetiapine 150mg modified-release tablets	Quetiapine Fumarate
44326	Seroquel XL 150mg tablets (AstraZeneca UK Ltd)	Quetiapine fumarate
45839	Quetiapine 25mg/5ml oral suspension	Quetiapine fumarate

46764	Quetiapine 12.5mg/5ml oral solution	Quetiapine Fumarate
46871	Quetiapine 12.5mg/5ml oral suspension	Quetiapine fumarate
49696	Quetiapine 25mg/5ml oral solution	Quetiapine fumarate
51178	Quetiapine 50mg/5ml oral suspension	Quetiapine fumarate
53552	Quetiapine 25mg tablets (Zentiva)	Quetiapine fumarate
54483	Sondate XL 300mg tablets (Teva UK Ltd)	Quetiapine fumarate
55870	Quetiapine oral liquid	Quetiapine Fumarate
56647	Quetiapine 300mg tablets (Arrow Generics Ltd)	Quetiapine fumarate
57034	Sondate XL 200mg tablets (Teva UK Ltd)	Quetiapine fumarate
57412	Sondate XL 400mg tablets (Teva UK Ltd)	Quetiapine fumarate
57612	Seroquel XL 400mg tablets (Lexon (UK) Ltd)	Quetiapine fumarate
57613	Seroquel XL 50mg tablets (Sigma Pharmaceuticals Plc)	Quetiapine fumarate
58821	Quetiapine 25mg tablets (Dr Reddy's Laboratories (UK) Ltd)	Quetiapine fumarate
58935	Tenprolide XL 400mg tablets (Actavis UK Ltd)	Quetiapine fumarate
59215	Quetiapine 150mg tablets (Ranbaxy (UK) Ltd)	Quetiapine fumarate
8881	Remoxipride 150mg capsule	Remoxipride Hydrochloride Monohydrate
12445	Remoxipride 300mg capsule	Remoxipride Hydrochloride Monohydrate
16223	Roxiam 300mg Capsule (AstraZeneca UK Ltd)	Remoxipride Hydrochloride Monohydrate
19016	Roxiam 150mg Capsule (AstraZeneca UK Ltd)	Remoxipride Hydrochloride Monohydrate
23034	Remoxipride 75mg capsule	Remoxipride Hydrochloride Monohydrate
48077	Roxiam ir 75mg Capsule (AstraZeneca UK Ltd)	Remoxipride Hydrochloride Monohydrate
302	Risperidone 1mg/ml oral solution sugar free	Risperidone
631	Risperdal 500microgram tablets (Janssen-Cilag Ltd)	Risperidone
667	Risperidone 500microgram tablets	Risperidone
1320	Risperidone 1mg tablets	Risperidone
1321	Risperidone 2mg tablets	Risperidone
2786	Risperidone 6mg tablets	Risperidone
2787	Risperidone 4mg tablets	Risperidone
4820	Risperdal 1mg tablets (Janssen-Cilag Ltd)	Risperidone
5219	Risperidone 3mg tablets	Risperidone
5262	Risperdal 1mg/ml liquid (Janssen-Cilag Ltd)	Risperidone
6373	Risperidone 1mg orodispersible tablets sugar free	Risperidone
7382	Risperidone 500microgram orodispersible tablets sugar free	Risperidone
9340	Risperdal 3mg tablets (Janssen-Cilag Ltd)	Risperidone

9475	Risperdal 4mg tablets (Janssen-Cilag Ltd)	Risperidone
9659	Risperdal 2mg tablets (Janssen-Cilag Ltd)	Risperidone
11799	Risperdal 6mg tablets (Janssen-Cilag Ltd)	Risperidone
11821	Risperdal Quicklet 500microgram orodispersible tablets (Janssen-Cilag Ltd)	Risperidone
11828	Risperidone 2mg orodispersible tablets sugar free	Risperidone
14767	Risperdal Consta 50mg powder and solvent for suspension for injection vials (Janssen-Cilag Ltd)	Risperidone
14789	Risperdal Consta 37.5mg powder and solvent for suspension for injection vials (Janssen-Cilag Ltd)	Risperidone
16006	Risperdal Quicklet 2mg orodispersible tablets (Janssen-Cilag Ltd)	Risperidone
16425	Risperidone 37.5mg powder and solvent for suspension for injection vials	Risperidone
16434	Risperidone 25mg powder and solvent for suspension for injection vials	Risperidone
16489	Risperidone 50mg powder and solvent for suspension for injection vials	Risperidone
16908	Risperdal Consta 25mg powder and solvent for suspension for injection vials (Janssen-Cilag Ltd)	Risperidone
16986	Risperdal Quicklet 1mg orodispersible tablets (Janssen-Cilag Ltd)	Risperidone
35141	Risperidone 3mg orodispersible tablets sugar free	Risperidone
35548	Risperdal Quicklet 3mg orodispersible tablets (Janssen-Cilag Ltd)	Risperidone
35589	Risperidone 4mg orodispersible tablets sugar free	Risperidone
35953	Risperdal Quicklet 4mg orodispersible tablets (Janssen-Cilag Ltd)	Risperidone
46677	Risperidone 500microgram tablets (Actavis UK Ltd)	Risperidone
47832	Risperidone 500microgram tablets (A A H Pharmaceuticals Ltd)	Risperidone
51240	Risperidone 125micrograms/5ml oral solution	Risperidone
51444	Risperdal Consta 50mg powder and solvent for suspension for injection vials (Waymade Healthcare Plc)	Risperidone
54346	Risperidone 1mg tablets (Teva UK Ltd)	Risperidone
55661	Risperidone 1mg tablets (Kent Pharmaceuticals Ltd)	Risperidone
56387	Risperidone 1mg/ml oral solution sugar free (Alliance Healthcare (Distribution) Ltd)	Risperidone
57217	Risperidone 1mg tablets (Generics (UK) Ltd)	Risperidone
58822	Risperidone 4mg tablets (Almus Pharmaceuticals Ltd)	Risperidone
59548	Risperidone 2mg tablets (Alliance Healthcare (Distribution) Ltd)	Risperidone
59829	Risperidone 3mg tablets (A A H Pharmaceuticals Ltd)	Risperidone
12666	Sertindole 4mg tablets	Sertindole
16998	Sertindole 12mg tablets	Sertindole
17050	Sertindole 20mg tablets	Sertindole
19900	Sertindole 16mg tablets	Sertindole
23162	Serdolect 16mg tablets (Lundbeck Ltd)	Sertindole
25966	Serdolect 4mg tablets (Lundbeck Ltd)	Sertindole

31063	Serdolect 12mg tablets (Lundbeck Ltd)	Sertindole
2135	Sulpiride 200mg tablets	Sulpiride
8903	Sulpiride 200mg/5ml oral solution sugar free	Sulpiride
9247	Sulpiride 400mg tablets	Sulpiride
10666	Dolmatil 200mg tablets (Sanofi)	Sulpiride
18181	Sulpor 200mg/5ml oral solution (Rosemont Pharmaceuticals Ltd)	Sulpiride
18352	Sulpitil 200mg tablets (Pfizer Ltd)	Sulpiride
24053	Sulparex 200mg Tablet (E R Squibb and Sons Ltd)	Sulpiride
24069	Dolmatil 400mg tablets (Sanofi)	Sulpiride
34810	Sulpiride 200mg tablets (Wockhardt UK Ltd)	Sulpiride
41675	Sulpiride 200mg tablets (IVAX Pharmaceuticals UK Ltd)	Sulpiride
43423	Sulpiride 200mg tablets (A A H Pharmaceuticals Ltd)	Sulpiride
43522	Sulpiride 200mg tablets (Teva UK Ltd)	Sulpiride
1192	Thioridazine 10mg tablets	Thioridazine hydrochloride
1218	Thioridazine 25mg tablets	Thioridazine hydrochloride
1314	Thioridazine 50mg tablets	Thioridazine hydrochloride
2801	Thioridazine 10mg/5ml Oral solution	Thioridazine Hydrochloride
3021	Thioridazine 100mg tablets	Thioridazine hydrochloride
3605	Thioridazine 25mg/5ml oral solution	Thioridazine hydrochloride
10405	Thioridazine 25mg/5ml sugar free Oral solution	Thioridazine Hydrochloride
15598	Thioridazine 100mg/5ml sugar free Oral solution	Thioridazine Hydrochloride
17399	Thioridazine 50mg/5ml Oral solution	Thioridazine Hydrochloride
34905	Thioridazine 25mg Tablet (IVAX Pharmaceuticals UK Ltd)	Thioridazine hydrochloride
35787	Thioridazine 50mg Tablet (IVAX Pharmaceuticals UK Ltd)	Thioridazine hydrochloride
42816	Thioridazine 50mg/5ml Oral solution (Rosemont Pharmaceuticals Ltd)	Thioridazine Hydrochloride
45860	Thioridazine 100mg Tablet (IVAX Pharmaceuticals UK Ltd)	Thioridazine hydrochloride
47361	Thioridazine 10mg/5ml Oral solution (Rosemont Pharmaceuticals Ltd)	Thioridazine Hydrochloride
1245	Trifluoperazine 5mg tablets	Trifluoperazine hydrochloride
1316	Stelazine 5mg tablets (Mercury Pharma Group Ltd)	Trifluoperazine hydrochloride
1318	Stelazine 1mg tablets (Mercury Pharma Group Ltd)	Trifluoperazine hydrochloride
1857	Trifluoperazine 1mg tablets	Trifluoperazine hydrochloride
3356	Parstelin Tablet (GlaxoSmithKline Consumer Healthcare)	Trifluoperazine Hydrochloride
3955	Tranylcypromine with trifluoperazine Tablet	Trifluoperazine Hydrochloride
7479	Stelazine 1mg/ml Injection (Goldshield Pharmaceuticals Ltd)	Trifluoperazine Hydrochloride
8537	Trifluoperazine 1mg/ml Injection	Trifluoperazine Hydrochloride
8985	Stelazine 1mg/5ml syrup (Mercury Pharma Group Ltd)	Trifluoperazine hydrochloride

11531	Trifluoperazine 5mg/5ml oral solution sugar free	Trifluoperazine hydrochloride
13145	Trifluoperazine 1mg/5ml oral solution sugar free	Trifluoperazine hydrochloride
18289	Stelazine 10mg/ml Concentrate (Goldshield Pharmaceuticals Ltd)	Trifluoperazine Hydrochloride
18668	Trifluoperazine 10mg/ml concentrate	Trifluoperazine Hydrochloride
24890	Trifluoperazine with tranylcypromine 1mg + 10mg Tablet	Trifluoperazine Hydrochloride
29948	Stelazine Forte 1mg/ml oral solution (Mercury Pharma Group Ltd)	Trifluoperazine hydrochloride
40162	Trifluoperazine 1mg tablets (A A H Pharmaceuticals Ltd)	Trifluoperazine hydrochloride
41663	Trifluoperazine 5mg tablets (A A H Pharmaceuticals Ltd)	Trifluoperazine hydrochloride
21027	Triperidol 1mg Tablet (Lagap)	Trifluperidol
21047	Triperidol 0.5mg Tablet (Lagap)	Trifluperidol
22814	Trifluperidol 1mg Tablet	Trifluperidol
23659	Trifluperidol 0.5mg Tablet	Trifluperidol
9515	Zoleptil 50 tablets (Movianto UK Ltd)	Zotepine
17504	Zotepine 25mg tablets	Zotepine
25336	Zotepine 50mg tablets	Zotepine
28759	Zotepine 100mg tablets	Zotepine
30088	Zoleptil 25 tablets (Movianto UK Ltd)	Zotepine
5762	Clopixol acuphase 50mg/ml Oily injection (Lundbeck Ltd)	Zuclopenthixol Acetate
14576	Zuclopenthixol acetate 50mg/ml oily injection	Zuclopenthixol Acetate
24270	Zuclopenthixol acetate 50mg/1ml solution for injection ampoules	Zuclopenthixol acetate
31537	Zuclopenthixol acetate 100mg/2ml solution for injection ampoules	Zuclopenthixol acetate
31538	Clopixol Acuphase 100mg/2ml solution for injection ampoules (Lundbeck Ltd)	Zuclopenthixol acetate
36101	Clopixol Acuphase 50mg/1ml solution for injection ampoules (Lundbeck Ltd)	Zuclopenthixol acetate
3774	Clopixol 200mg/ml Oily injection (Lundbeck Ltd)	Zuclopenthixol Decanoate
3775	Zuclopenthixol decanoate 200mg/ml oily injection	Zuclopenthixol Decanoate
12073	Clopixol Conc 500mg/1ml solution for injection ampoules (Lundbeck Ltd)	Zuclopenthixol decanoate
12224	Zuclopenthixol decanoate 500mg/1ml solution for injection ampoules	Zuclopenthixol decanoate
22049	Clopixol 200mg/1ml solution for injection ampoules (Lundbeck Ltd)	Zuclopenthixol decanoate
28355	Zuclopenthixol decanoate 200mg/1ml solution for injection ampoules	Zuclopenthixol decanoate
1319	Clopixol 10mg tablets (Lundbeck Ltd)	Zuclopenthixol dihydrochloride
9347	Clopixol 25mg tablets (Lundbeck Ltd)	Zuclopenthixol dihydrochloride

9686	Zuclopenthixol 10mg tablets	Zuclopenthixol dihydrochloride
12707	Zuclopenthixol 2mg tablets	Zuclopenthixol dihydrochloride
13368	Clopixol 2mg tablets (Lundbeck Ltd)	Zuclopenthixol dihydrochloride
13600	Zuclopenthixol 25mg tablets	Zuclopenthixol dihydrochloride
50214	Olanzapine 5mg orodispersible tablets	
51558	Amisulpride 12.5mg/5ml oral suspension	
52050	Haloperidol 1.5mg/5ml oral suspension	
52076	Amisulpride 12.5mg/5ml oral solution	
52940	Sondate XL 50mg tablets (Teva UK Ltd)	
55622	Olanzapine 10mg orodispersible tablets	
56143	Olanzapine 15mg orodispersible tablets	
56215	Quetiapine 50mg/5ml oral solution	
58067	Quetiapine 125mg/5ml oral suspension	
58425	Seroquel XL 50mg tablets (DE Pharmaceuticals)	
58936	Tenprolide XL 50mg tablets (Actavis UK Ltd)	
