# Supplementary material for: Antipsychotic Prescribing to Patients Diagnosed with Dementia Without a Diagnosis of Psychosis in the Context of National Guidance and Drug Safety Warnings: Longitudinal Study in UK General Practice
Source: Drug Saf. 2017 Apr 24;40(8):679–92. doi: 10.1007/s40264-017-0538-x (PMC5519656; doi:10.1007/s40264-017-0538-x)
Supplement: Supplementary file 2 — Electronic Supplementary Material 2: Description of population, sensitivity and other additional analyses (PDF 421 kb) [file 40264_2017_538_MOESM2_ESM.pdf]

Supplementary Appendix 2.

Antipsychotic prescribing to patients diagnosed with dementia in the context of national guidance and drug safety warnings: longitudinal study in UK general practice

S Jill Stocks, Evangelos Kontopantelis, Roger T Webb, Anthony J Avery, Alistair Burns, Darren M Ashcroft
Correspondence to Dr Jill Stocks; NIHR Greater Manchester Primary Care Patient Safety Translational Research Centre, Centre for Primary Care, Division of Population Health, Health Services Research and Primary Care, School of Health Sciences, University of Manchester, Manchester M13 9PL, UK
jill.stocks@manchester.ac.uk

Supplementary Results: Temporal changes in the study population from 2001 to 2014

Over the 14 year study period the annual number of patients in CPRD ranged from 4 to 5.9 million of which 3.5 to 4.8 million were deemed by CPRD to be of research quality. The proportion of patients aged 65 or over increased from 15.5% to 16.9%. For patients aged 65 or over, the prevalence of dementia increased from 1.9% in 2001 to 4.3% (Fig A) whereas the incidence of dementia remained fairly constant. In 2001, 12.5% of patients aged 65 or over with a dementia diagnosis also had a recorded diagnosis of psychosis which had declined to 6.5% by 2014 (Fig B).

Supplementary Results: Sensitivity analysis (Table A)

Repeating the before and after analysis including only the practices contributing data to CPRD for the entire period 2001 to 2014 had a negligible effect on the prevalence ratios. Including all patients with dementia (with or without a psychosis diagnosis) did not substantially alter the prevalence ratios.

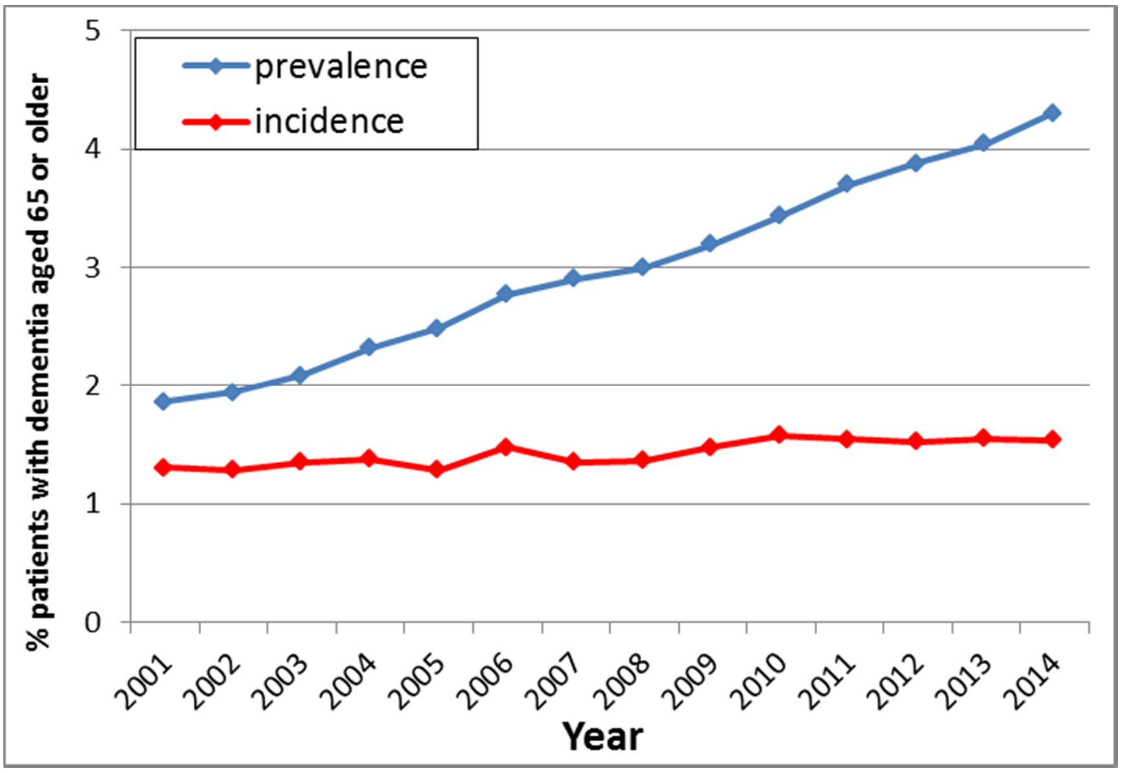

Figure A. Temporal changes in the proportion of patients with a diagnosis of dementia/all patients aged 65 or over currently registered with a CPRD practice.

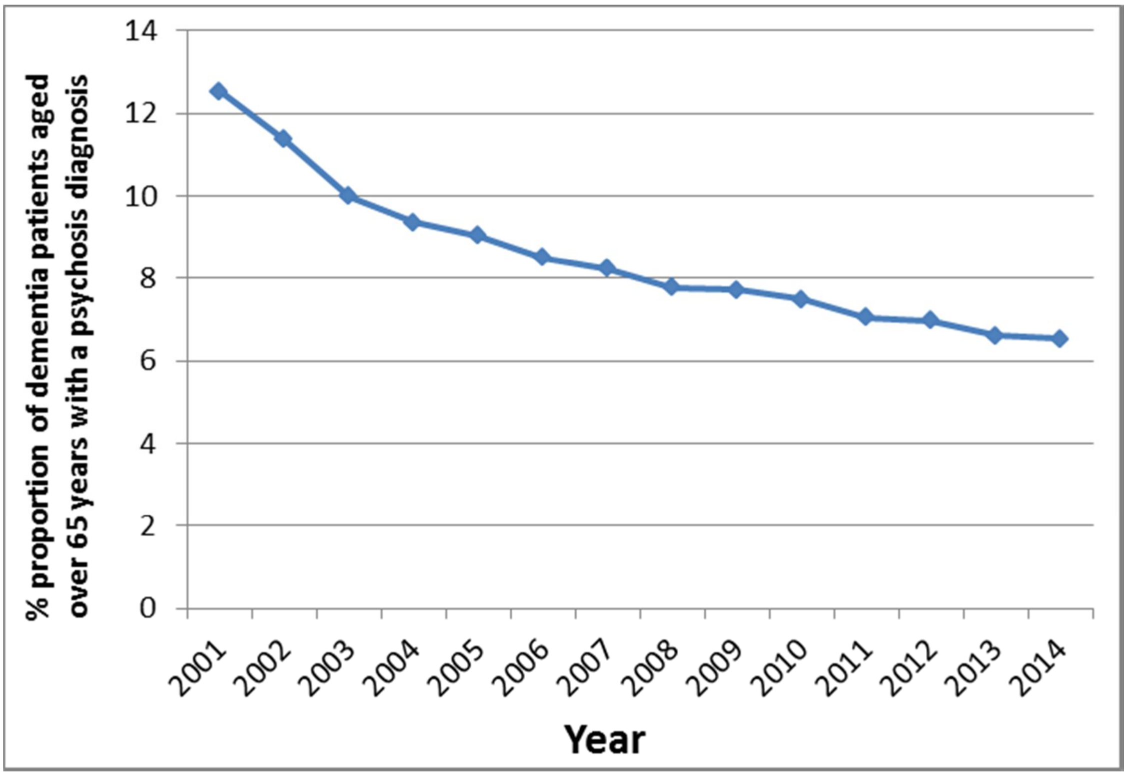

Figure B. Temporal changes in the proportion of patients with a diagnosis of psychosis/ patients aged 65 or over with a diagnosis of dementia recorded in CPRD.

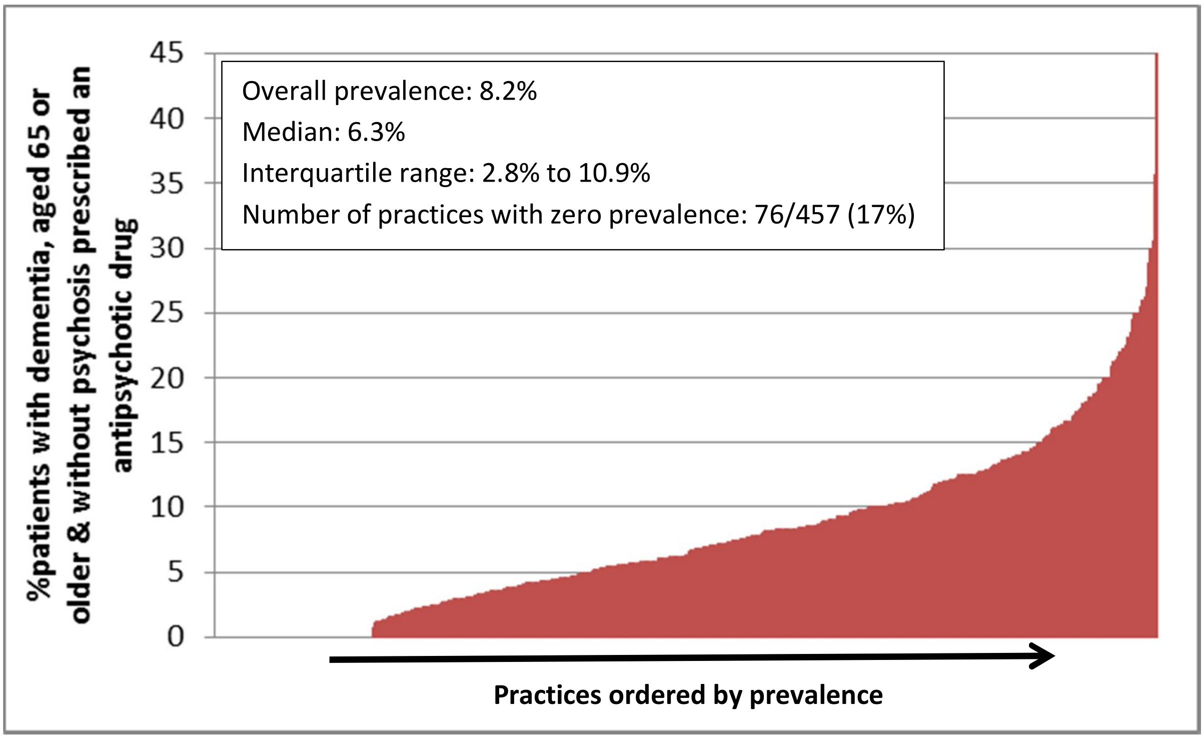

Figure C. Proportion of patients with dementia without a psychosis diagnosis prescribed antipsychotic drugs at least twice between 1st January 2014 and 30th June 2014 with at least three months between the first and last prescription by practice.

Table A. Sensitivity analyses: Prevalence ratios before and after each intervention adjusted for age, gender and clustering within practices

Drug type	Prevalence ratio: before and after intervention(95% CI)[1]		
	Main analysis – patients aged 65 years or older diagnosed with dementia without psychosis (639 practices)	Repeat analysis only including practices contributing to CPRD continuously from 2003 to 2012 (462 practices)	Repeat analysis including all patients aged 65 years or older diagnosed with dementia (with or without psychosis)
MHRA Committee for the Safety of Medicines warning Mar 2004			
All antipsychotics	0.81 (0.76 to 0.87)	0.81 (0.75 to 0.87)	0.81 (0.76 to 0.86)
1st generation	1.17 (1.03 to 1.31)	1.17 (1.02 to 1.35)	1.16 (1.04 to 1.30)
2nd generation	0.66 (0.61 to 0.71)	0.66 (0.61 to 0.72)	0.66 (0.62 to 0.71)
risperidone	0.48 (0.43 to 0.53)	0.49 (0.44 to 0.55)	0.48 (0.43 to 0.52)
olanzapine	0.60 (0.51 to 0.70)	0.59 (0.49 to 0.71)	0.63 (0.54 to 0.72)
quetiapine	1.51 (1.24 to 1.83)	1.53 (1.23 to 1.92)	1.49 (1.25 to 1.79)
amisulpride	1.47 (1.00 to 2.15)	1.84 (1.06 to 3.19)	1.56 (1.05 to 2.30)
NICE guidelines Nov 2006			
All antipsychotics	1.07 (1.01 to 1.13)	1.07 (1.00 to 1.14)	1.07 (1.02 to 1.13)
1st generation	1.02 (0.93 to 1.12)	1.01 (0.90 to 1.13)	1.02 (0.93 to 1.11)
2nd generation	1.08 (1.01 to 1.15)	1.08 (1.00 to 1.17)	1.08 (1.02 to 1.15)
risperidone	0.87 (0.74 to 1.03)	0.88 (0.73 to 1.05)	0.90 (0.78 to 1.04)
olanzapine	0.97 (0.80 to 1.17)	0.95 (0.77 to 1.17)	1.00 (0.85 to 1.18)
quetiapine	1.14 (1.05 to 1.24)	1.15 (1.05 to 1.27)	1.14 (1.05 to 1.23)
amisulpride	1.10 (0.92 to 1.31)	1.13 (0.92 to 1.40)	1.11 (0.94 to 1.30)
MHRA Drug Safety Updates Mar&Jun09, Expert review Oct09, Government pledge Nov09			
All antipsychotics	0.83 (0.79 to 0.88)	0.83 (0.78 to 0.88)	0.84 (0.80 to 0.88)
1st generation	0.85 (0.77 to 0.95)	0.86 (0.77 to 0.97)	0.84 (0.77 to 0.93)
2nd generation	0.84 (0.79 to 0.90)	0.84 (0.78 to 0.90)	0.85 (0.80 to 0.90)
risperidone	0.73 (0.61 to 0.87)	0.73 (0.60 to 0.88)	0.76 (0.65 to 0.90)
olanzapine	0.81 (0.66 to 1.00)	0.81 (0.64 to 1.02)	0.81 (0.68 to 0.96)
quetiapine	0.87 (0.81 to 0.94)	0.87 (0.80 to 0.94)	0.88 (0.82 to 0.94)
amisulpride	0.92 (0.78 to 1.09)	0.89 (0.74 to 1.08)	0.95 (0.81 to 1.10)
National Dementia Challenge Mar 2012, MHRA Drug Safety Update May 2012			
All antipsychotics	0.86 (0.81 to 0.92)	0.86 (0.81 to 0.93)	0.88 (0.84 to 0.93)
1st generation	0.81 (0.71 to 0.91)	0.81 (0.71 to 0.93)	0.84 (0.75 to 0.94)
2nd generation	0.89 (0.83 to 0.95)	0.90 (0.84 to 0.96)	0.91 (0.85 to 0.96)
risperidone	1.13 (0.99 to 1.29)	1.13 (0.98 to 1.29)	1.14 (1.01 to 1.29)
olanzapine	0.93 (0.76 to 1.14)	0.94 (0.76 to 1.17)	0.93 (0.78 to 1.10)
quetiapine	0.80 (0.74 to 0.88)	0.81 (0.74 to 0.89)	0.83 (0.76 to 0.90)
amisulpride	0.80 (0.64 to 0.99)	0.81 (0.64 to 1.01)	0.79 (0.65 to 0.97)

[1]prevalence ratio compares repeated prescribing for three months or longer during the six months after the intervention relative to the six months leading up to the intervention as defined in Table 1 in the main paper
