# Supplementary material for: Antipsychotic Prescribing to Patients Diagnosed with Dementia Without a Diagnosis of Psychosis in the Context of National Guidance and Drug Safety Warnings: Longitudinal Study in UK General Practice
Source: Drug Saf. 2017 Apr 24;40(8):679–92. doi: 10.1007/s40264-017-0538-x (PMC5519656; doi:10.1007/s40264-017-0538-x)

Supplementary Appendix 3.

Antipsychotic prescribing to patients diagnosed with dementia in the context of national guidance and drug safety warnings: longitudinal study in UK general practice

S Jill Stocks, Evangelos Kontopantelis, Roger T Webb, Anthony J Avery, Alistair Burns, Darren M Ashcroft
Correspondence to Dr Jill Stocks; NIHR Greater Manchester Primary Care Patient Safety Translational Research Centre, Centre for Primary Care, Division of Population Health, Health Services Research and Primary Care, School of Health Sciences, University of Manchester, Manchester M13 9PL, UK jill.stocks@manchester.ac.uk

Interrupted time series plots corresponding to Table 2 by class and individual antipsychotic drug

A. The MHRA Committee for Safety of Medicines warning: March 2004

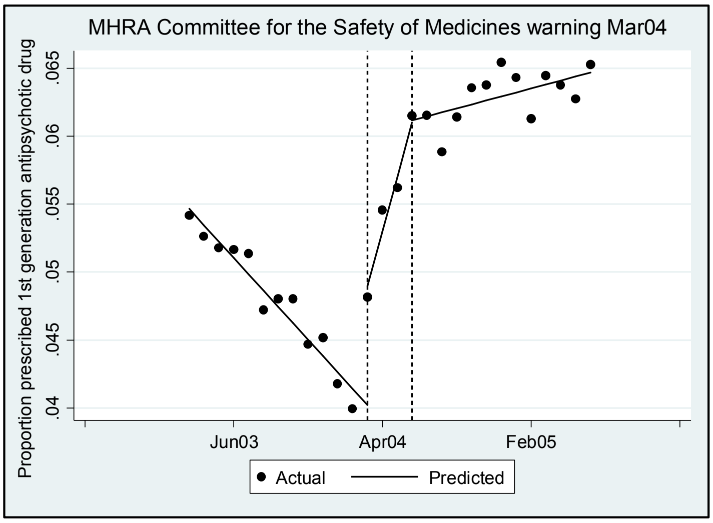

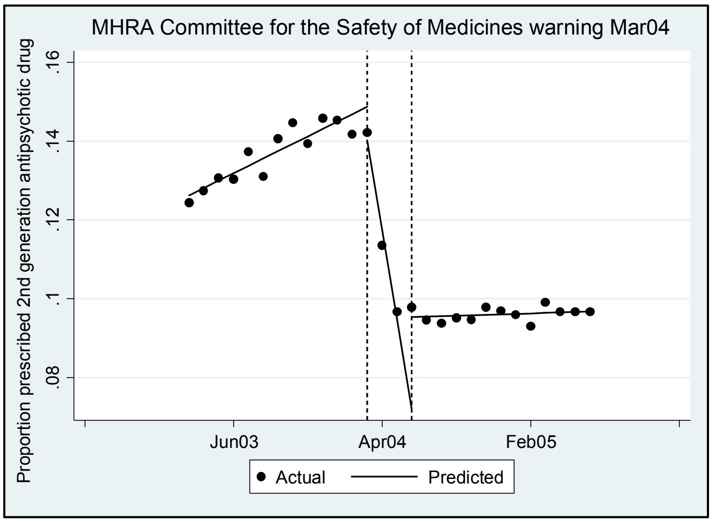

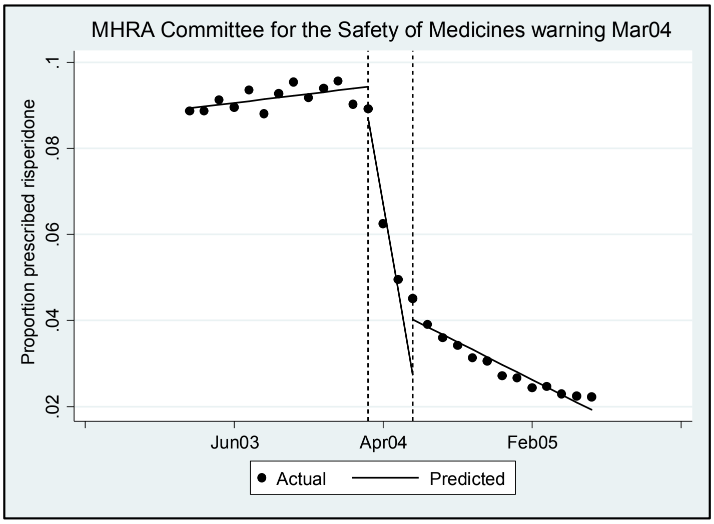

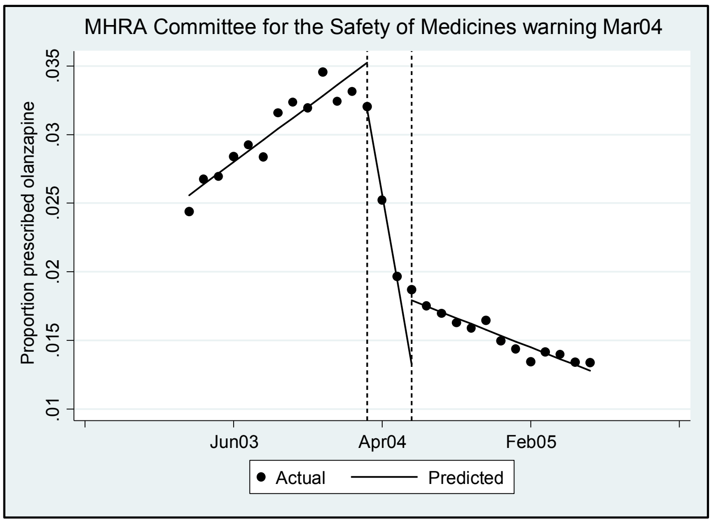

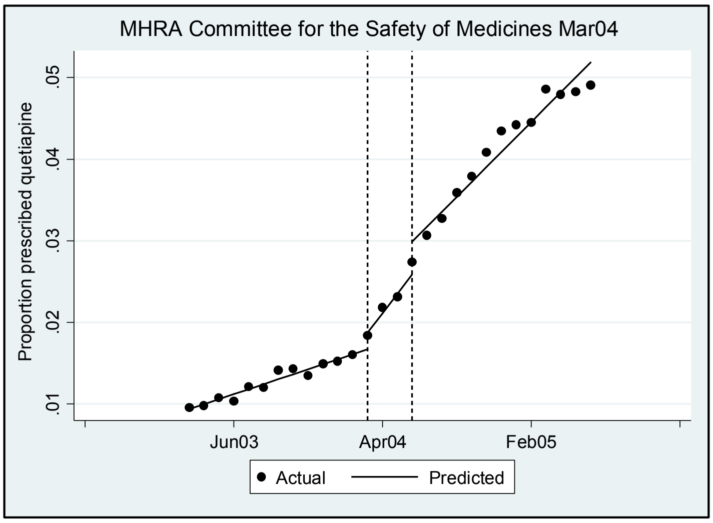

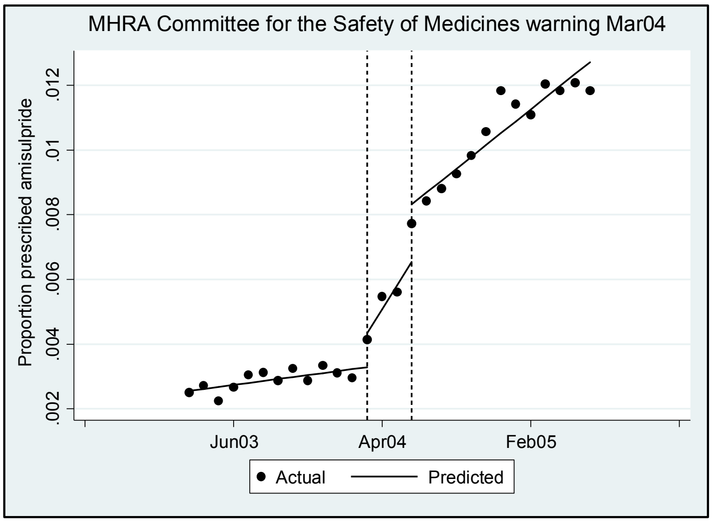

B. Publication of NICE guidelines: November 2006

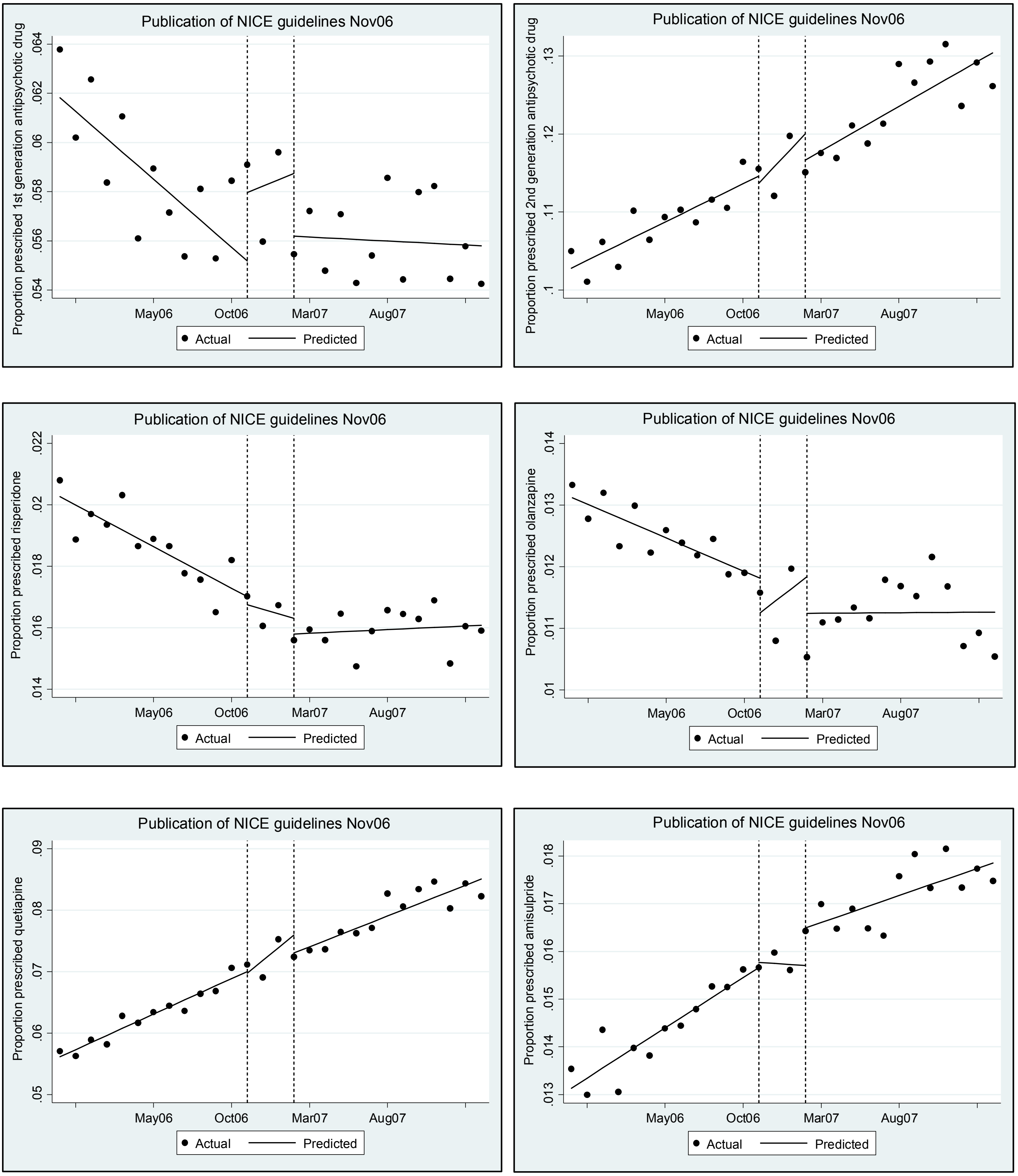

C. MHRA Drug Safety Updates: March & June 2009; Expert review: October 2009; Government pledge: November 2009

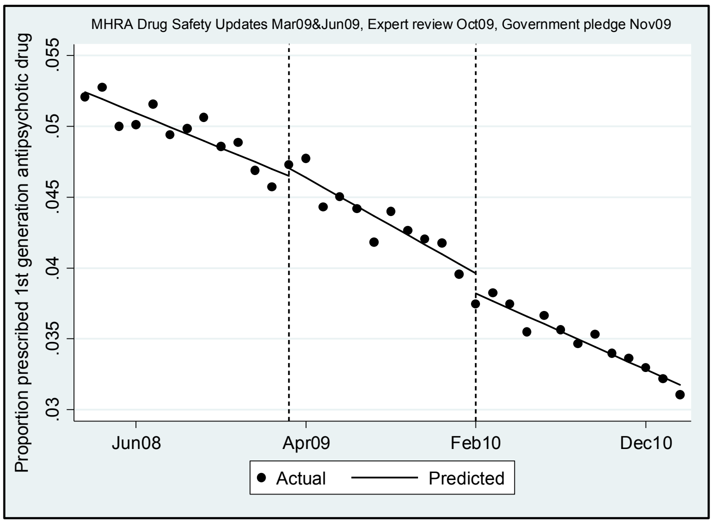

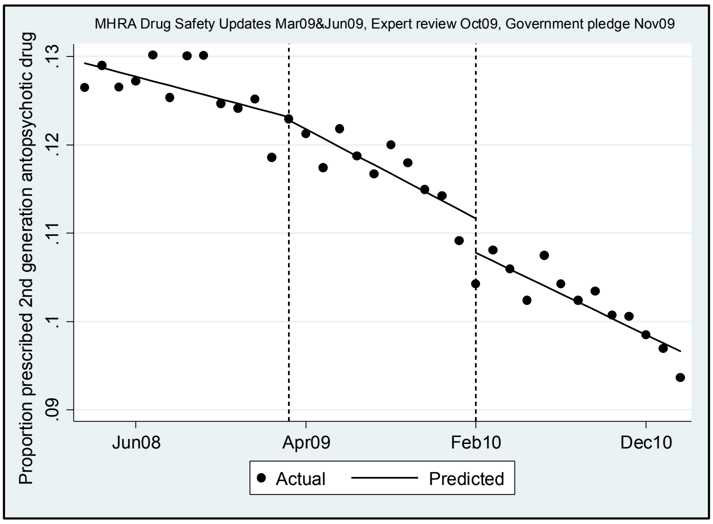

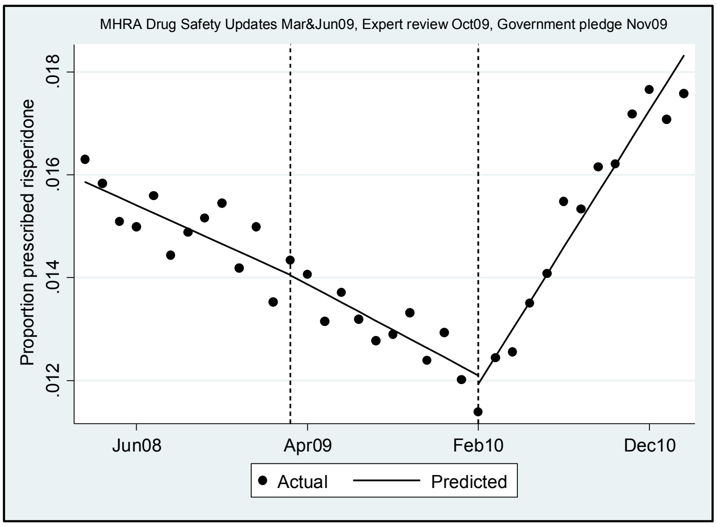

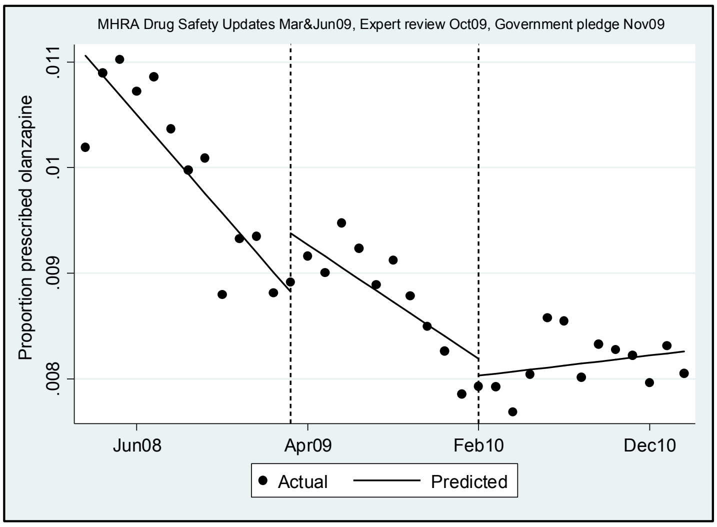

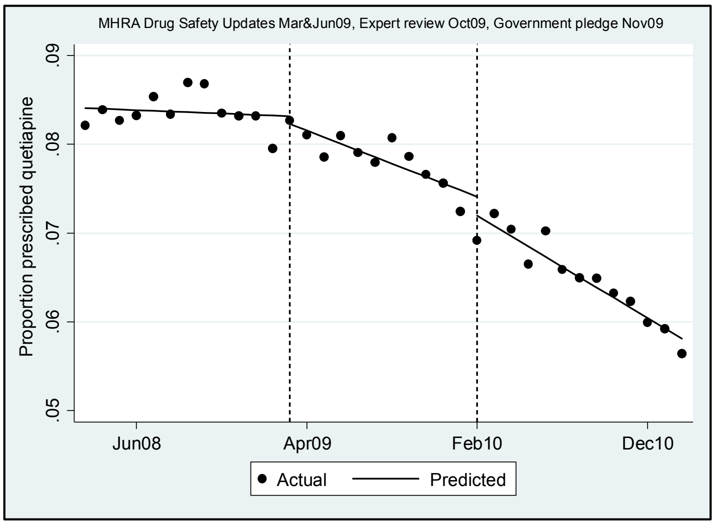

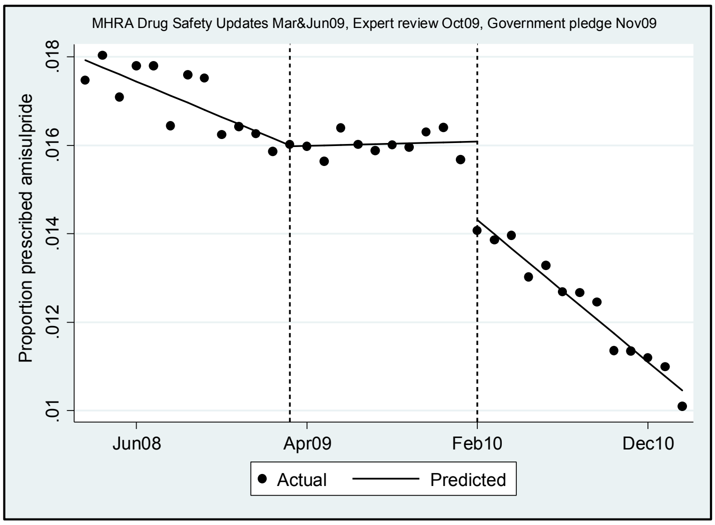

D. National Dementia Challenge: March 2012; MHRA Drug Safety Update: May2012

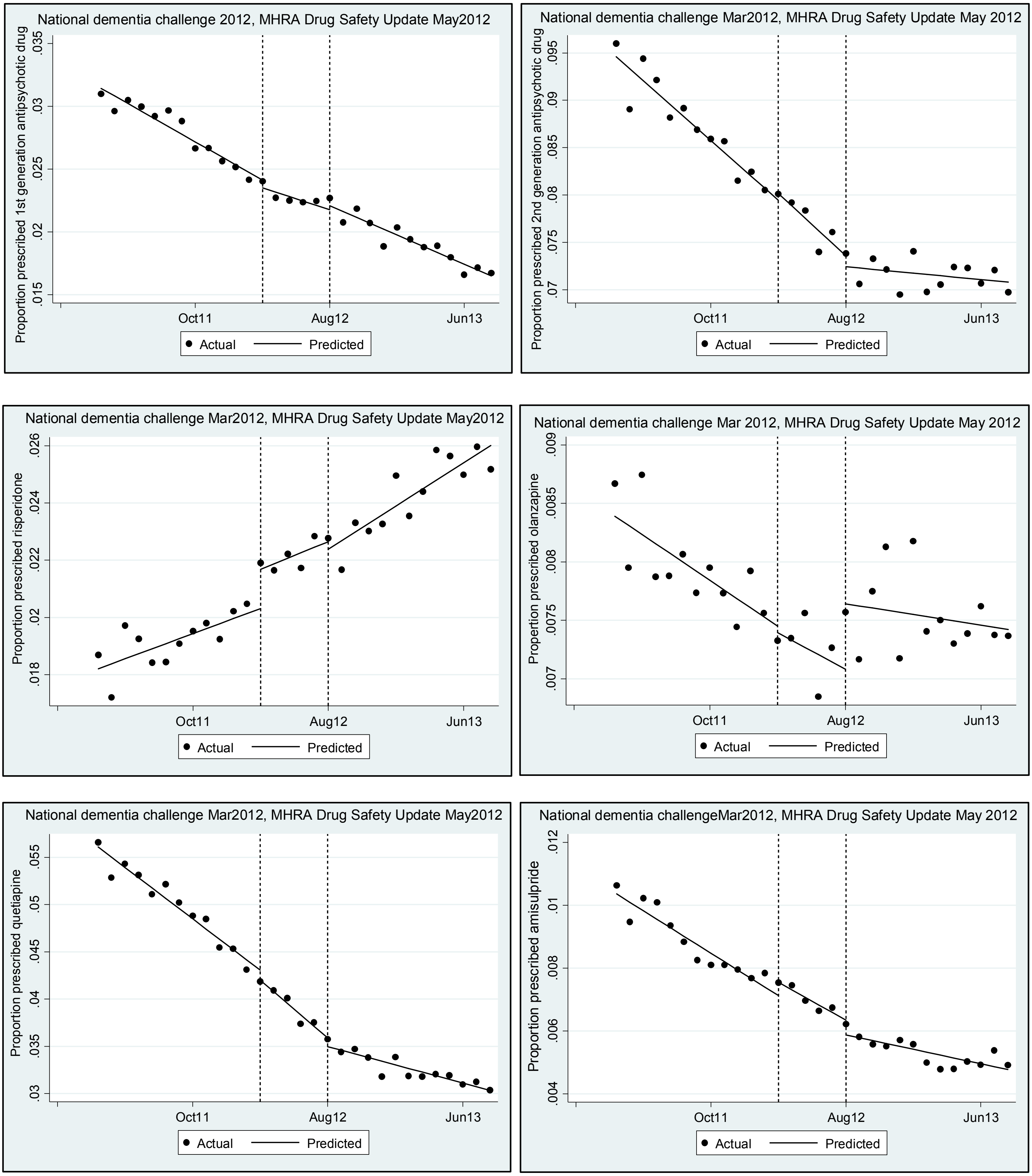

Supplement: Supplementary file 3 — Electronic Supplementary Material 3: Interrupted time series plots by class and individual antipsychotic drug (PDF 197 kb) [file 40264_2017_538_MOESM3_ESM.pdf]
